# A single‐molecule platform for photo‐triggered peroxynitrite release with built‐in fluorescence calibration

**DOI:** 10.1002/smo2.70013

**Published:** 2025-08-14

**Authors:** Mengqi Liu, Yufeng Zhu, Xiaoyue Zhang, Yanyan Hou, Huan Zhang, Francisco Galindo, Youjun Yang, Xuhong Qian, Xiao Luo

**Affiliations:** ^1^ State Key Laboratory of Bioreactor Engineering Shanghai Key Laboratory of Chemical Biology School of Pharmacy East China University of Science and Technology Shanghai China; ^2^ Shanghai Engineering Research Center of Molecular Therapeutics and New Drug Development, School of Chemistry and Molecular Engineering, East China Normal University Shanghai China; ^3^ Departamento de Química Inorgánica y Orgánica Universitat Jaume I de Castellón Castellón de la Plana Spain

**Keywords:** fluorescence, oxidative stress, peroxynitrite, photo‐triggered, rhodamine

## Abstract

Peroxynitrite (ONOO^−^) is central to both physiological signaling and diverse pathological processes. Its dual nature underscores the need for precise tools to investigate its spatiotemporal dynamics and biological functions. However, the controlled generation and real‐time tracking of ONOO^−^ remain challenging due to its short half‐life and high reactivity. Current small‐molecule ONOO^−^ donors often suffer from limitations such as slow release, low efficiency, and off‐target effects. To overcome these challenges, here we report a new class of photo‐triggered ONOO^−^ donors (**O‐PND** and **Si‐PND**) based on a single rhodamine‐derived scaffold, enabling precise ONOO^−^ release with built‐in fluorescence calibration. These molecular tools facilitate efficient ONOO^−^ generation under blue light irradiation, as confirmed in PBS and live cells, and exhibit excellent cell membrane permeability. Upon intracellular activation, **O‐PND** and **Si‐PND** induced a marked increase in oxidative stress. However, further studies reveal that the rapid transient ONOO^−^ burst in RAW264.7 cells was insufficient to significantly modulate macrophage polarization. Collectively, these robust self‐reporting ONOO^−^ donors provide a powerful single‐molecule platform for investigating ONOO^−^‐mediated biological mechanisms with spatiotemporal precision.

## Graphical abstract

A new class of photoactivatable peroxynitrite (ONOO^−^) donors based on a single rhodamine scaffold enables spatiotemporally controlled ONOO^−^ release with fluorescence calibration, offering a powerful tool for probing ONOO^−^ biology and redox signaling in live cells.

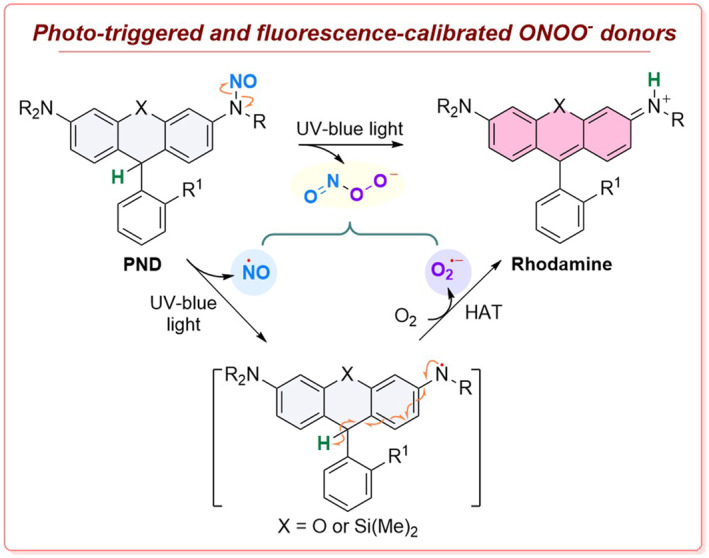

## 1 INTRODUCTION

Peroxynitrite (ONOO^−^) is a highly reactive nitrogen species generated through the diffusion‐controlled reaction between nitric oxide (NO) and superoxide anion (O_2_^•–^).^[1]^ Owing to its strong oxidative and nitrative power, ONOO^−^ plays a pivotal role in both physiological signaling and the pathogenesis of various diseases.^[2]^ At basal levels it supports essential redox signaling but excessive accumulation can induce significant oxidative damage to cellular components, including lipids, proteins, and nucleic acids.^[3]^ Such damage has been implicated in a broad spectrum of diseases, such as neurodegenerative disorders, cardiovascular diseases, chronic inflammation, and cancer.^[4]^ The dualistic nature of ONOO^−^—functioning as both a regulatory mediator and a cytotoxic effector—highlights the urgent need for precise tools capable of controlling its generation and monitoring its dynamics with spatiotemporal resolution. However, the intrinsic challenges posed by its extremely short half‐life (less than 10 ms under physiological conditions) and high reactivity continue to impede efforts to accurately probe its biological functions.^[5]^

**FIGURE 1:**
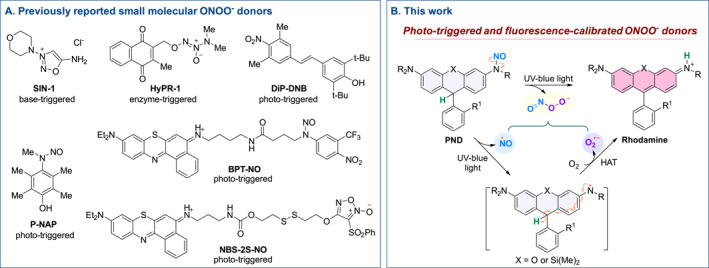
(a) Representative examples of previously reported small‐molecule ONOO^−^ donors. (b) Proposed photolysis mechanism of **PND**, illustrating its function as a photo‐triggered and fluorescence‐calibrated ONOO^−^ donor.

Current strategies for ONOO^−^ generation in biological systems fall into two broad categories: small‐molecule‐based donors and material‐based platforms. Material‐based approaches typically co‐encapsulate NO and O_2_^•–^ donors to enable sustained ONOO^−^ release for therapeutic purposes, harnessing its potent oxidative and nitrative activities. These strategies have been extensively applied in the treatment of bacterial infections and cancer as evidenced by numerous seminal studies reported in the literature.^[6]^ However, they often afford poor spatiotemporal control, asynchronous or mismatched release of NO and O_2_^•–^, and rely on chemically complex formulations. Such limitations pose challenges when probing the chemical biology of ONOO^−^ in a mechanistically precise manner. In contrast, small‐molecule ONOO^−^ donors are better suited for fundamental biological studies, owing to their well‐defined structures, tunable release kinetics, and facile application. Among these, SIN‐1 is one of the most commonly used ONOO^−^ donors.^[7]^ Yet, its slow and inefficient ONOO^−^ generation requires millimolar concentrations to achieve biologically relevant fluxes, often leading to off‐target effects. To overcome these drawbacks, various enzyme‐ or photo‐triggered ONOO^−^ donors, such as **HyPR‐1**,^[8]^ **Dip‐DNB**,^[9]^ **P‐NAP** ^[10]^ and **BPT‐NO** derivatives^[11]^ (Figure 1a), have been developed. These donors offer more controlled release profiles and improved targeting capabilities. Nonetheless, many of the existing photoactivatable donors still suffer from insufficient ONOO^−^ generation efficiency, lack robust fluorescence self‐calibration, or suboptimal stability, which hinder their broader application in real‐time biological investigations.

In this work, we designed and constructed a new class of robust molecular tools, that is, **O‐PND** and **Si‐PND**, capable of photo‐triggered ONOO^−^ release with integrated fluorescence calibration. These donors enable efficient ONOO^−^ generation under precise light control, with reliable self‐reporting features, thus providing a powerful platform for spatiotemporally resolved investigations of ONOO^−^‐mediated biological mechanisms. The photo‐triggered release of ONOO^−^ from **O‐PND** and **Si‐PND** was confirmed unambiguously in both PBS and live cells. These donors exhibit good cell membrane permeability, and intracellular activation led to a significant increase in oxidative stress. Further studies revealed that the rapid transient ONOO^−^ burst in RAW264.7 cells was insufficient to significantly modulate macrophage polarization.

## 2 RESULTS AND DISCUSSION

### 2.1 Molecular design and synthesis

A reduced form of rhodamine bearing an *N*‐nitrosated aniline moiety was designed as a photo‐triggered and fluorescence‐calibrated peroxynitrite donor (**PND**) (Figure 1b). Upon light irradiation, homolytic cleavage of the N–N bond in the nitrosamine group occurs, releasing NO and generating an aniline radical. This radical undergoes a hydrogen atom transfer (HAT)^[12]^ while concurrently reducing molecular oxygen to generate O_2_^•–^, thereby regenerating the stable rhodamine fluorophore. The in situ generated NO and O_2_^•–^ rapidly combine at a diffusion‐controlled rate to yield ONOO^−^ (Figure 1b). According to the above mechanism, the formation of ONOO^−^ is stoichiometrically coupled to that of the rhodamine fluorophore. Thus, the fluorescence signal of rhodamine can serve as a built‐in reporter for real‐time tracking of the release site and kinetics of ONOO^−^. Additionally, the rate and efficiency of release can be finely tuned by varying the intensity of the activating light.

**FIGURE 2:**
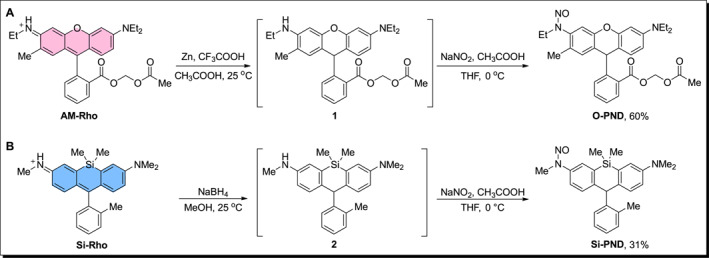
Synthesis of (a) **AM‐PND** and (b) **Si‐PND** for photo‐induced in situ generation of ONOO^−^.

Rhodamine and Si‐rhodamine were selected as two representative scaffolds to meet the requirements of multichannel imaging in practical applications, simultaneously demonstrating the general applicability of the proposed strategy. The general synthetic route for **O‐PND** and **Si‐PND** involved the initial preparation of their corresponding rhodamine fluorophores, that is, **AM‐Rho** and **Si‐Rho** (Figure 2). **AM‐Rho** was synthesized from rhodamine 6G through a four‐step reaction sequence (Scheme S1), while **Si‐Rho** was prepared starting from 3‐bromoaniline and 2‐bromo‐4‐fluorobenzaldehyde via a seven‐step synthetic route (Scheme S2). The resulting rhodamine scaffolds were subsequently reduced to their leuco forms using Zn powder or NaBH_4_ at room temperature. Finally, the aniline moiety was nitrosated using NaNO_2_ under cold acidic conditions to yield **O‐PND** (Figure 2a) and **Si‐PND** (Figure 2b) with overall yields of 60% and 31%, respectively.

### 2.2 Photo‐triggered generation of rhodamines from PND

**FIGURE 3:**
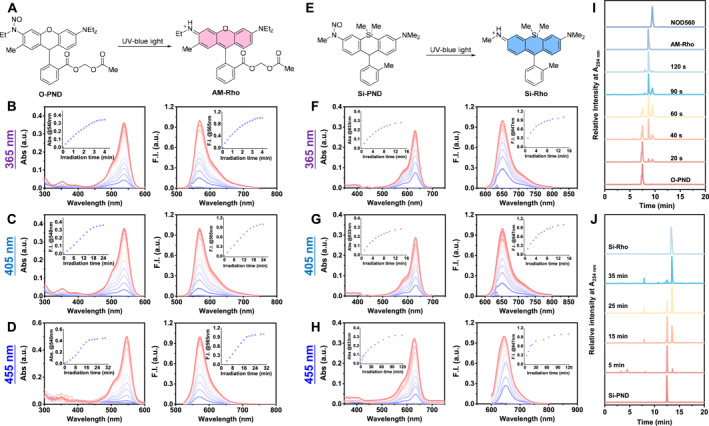
Spectral studies of **O‐PND** and **Si‐PND** photolysis by UV‐blue light and identification of their products. The proposed photolysis mechanism of (a) **O‐PND** and (e) **Si‐PND**. UV/Vis absorbance and fluorescence spectra of **O‐PND** (5 μM, in pH 7.4 PBS with 1% DMF) upon irradiation with (b) 365 nm (15 mW cm^−2^), (c) 405 nm (15 mW cm^−2^), or (d) 455 nm (20 mW cm^−2^) LED light. Insets: absorbance at 540 nm and fluorescence intensity at 565 nm as a function of irradiation time for panel 3B‐3D, respectively. UV/Vis absorbance and fluorescence spectra of **Si‐PND** (5 μM, in pH 7.4 PBS with 0.5% DMF) upon irradiation with (f) 365 nm (15 mW cm^−2^), (g) 405 nm (30 mW cm^−2^), or (h) 455 nm (20 mW cm^−2^) LED light. Insets: absorbance at 633 nm and fluorescence intensity at 647 nm as a function of time for panels 3G‐3I, respectively. (i) HPLC profiles of **O‐PND** photolysis showing the formation of **AM‐Rho**. (j) HPLC profiles of **Si‐PND** photolysis showing the formation of **Si‐Rho.**

The photolysis behaviors of **O‐PND** and **Si‐PND** were examined in PBS (10 mM, pH = 7.4) containing 1% DMF as a co‐solvent. Prior to light exposure, the absorption of **O‐PND** in the visible region beyond 350 nm was minimal, and no fluorescence was detected upon excitation at 540 nm. Upon irradiation with 365 nm LED light, a distinct absorption peak appeared at 540 nm, with absorbance increasing rapidly within the first 4 min before plateauing, likely due to the consumption of **O‐PND**. When the solution was excited at 540 nm, a fluorescence emission band with a peak at 565 nm was observed (Figure 3b). The kinetic profile of the fluorescence intensity increase mirrored the changes in the UV‐Vis absorption spectra (Figure 3b, insets). Both the absorption and emission band shapes and wavelengths were consistent with those of the expected rhodamine dye **AM‐Rho** (Figure 3a). To further confirm that **AM‐Rho** is the photolysis product of **O‐PND**, the photolytic process was monitored by HPLC analysis. A solution of **O‐PND** (0.5 mM) in PBS containing 5% MeCN was irradiated with a 365 nm LED (100 mW cm^−2^) and aliquots were analyzed (HPLC) at set intervals. As the irradiation time increased, the peak corresponding to **O‐PND** (retention time: 7.5 min) gradually decreased, while a new peak corresponding to **AM‐Rho** (retention time: 8.7 min) progressively emerged (Figure 3i). These results confirmed that light activation induces the transformation of **O‐PND** into **AM‐Rho**. It was noted that a small peak with a retention time around 9.5 min appeared along with the generation of **AM‐Rho** and eventually disappeared. This retention time was consistent with that of our previously reported NO donor, **NOD560**.^[13]^ We speculated that at a relatively high concentration of 0.5 mM, a portion of **O‐PND** was oxidized by the released ONOO^−^ into the corresponding NO donor. The photolysis spectral behaviors of **O‐PND** in response to 405 nm (Figure 3c) and 455 nm LED irradiation (Figure 3d) were similar to those observed under 365 nm light. However, as the activation wavelength increased, the time required for photolysis to reach a plateau was prolonged—from approximately 4 min under 365 nm to around 20 min under 455 nm irradiation. These results collectively confirmed that **O‐PND** can be efficiently converted to **AM‐Rho** under UV to blue light irradiation.

The photolytic conversion of **Si‐PND** to **Si‐Rho** was examined under the same conditions. Initially, **Si‐PND** exhibited negligible absorption in the visible region and no detectable fluorescence. Upon irradiation with 365 nm (Figure 3f), 405 nm (Figure 3g), or 455 nm (Figure 3h) LED light, characteristic absorption (*λ*_max_ = 633 nm) and fluorescence (*λ*_em_ = 647 nm) signals corresponding to **Si‐Rho** gradually emerged, indicating successful photoconversion (Figure 3e). The kinetic profiles revealed a slower photoreaction compared to **O‐PND**, requiring a longer irradiation time to reach the plateau phase at the same light intensity. HPLC analysis further confirmed the formation of **Si‐Rho** as the major photolysis product, with its retention time matching that of the independently synthesized reference compound **Si‐Rho** (Figure 3j).

### 2.3 Photo‐triggered release of ONOO^−^ from PND

**FIGURE 4:**
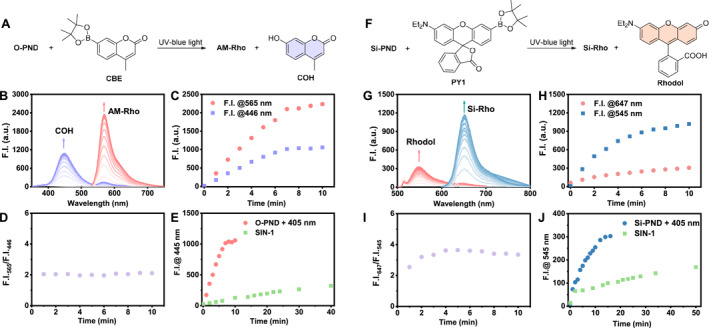
Probing ONOO^−^ with (a) **CBE** or (f) **PY1** during the photolysis of **O‐PND** or **Si‐PND**. (b) Fluorescence spectra of a mixture of **O‐PND** (5 μM) and CBE (5 μM) in PBS (pH 7.4 with 1% DMF) upon irradiation with a 405 nm LED (30 mW/cm^2^). (c) Fluorescence intensity at 446 nm (*λ*_ex_ = 325 nm) and 565 nm (*λ*_ex_ = 540 nm) as a function of irradiation time for panel 4B. (d) Intensity ratio of 565 versus 446 nm. (e) Comparation of **CBE** response to **O‐PND** photolysis or SIN‐1 (10 μM). (g) Fluorescence spectra of a mixture of **Si‐PND** (5 μM) and **PY1** (5 μM) in PBS (pH 7.4) upon irradiation with a 405 nm LED (100 mW/cm^2^). (h) Fluorescence intensity at 545 nm (*λ*_ex_ = 514 nm) and 647 nm (*λ*_ex_ = 633 nm) as a function of irradiation time for panel 4G. (i) Intensity ratio of 647 versus 545 nm. (j) Comparation of **PY1** response to **Si‐PND** photolysis or SIN‐1 (10 μM).

The photo‐triggered release of ONOO^−^ from **PND** was examined with **CBE**,^[14]^ a turn‐on probe based on a coumarin scaffold, emits in the blue channel upon reacting with ONOO^−^ (Figure 4a). A mixture of **PND** and **CBE** in PBS containing 1% DMF was irradiated with a 405 nm LED for various durations. Fluorescence emission spectra were recorded using excitation wavelengths of 446 and 565 nm, respectively. As the irradiation time increased, a gradual enhancement of fluorescence corresponding to the ONOO^−^‐induced product **COH** was observed at 446 nm (Figure 4b, purple lines; Figure 4c, purple dots). Simultaneously, **O‐PND** underwent photolysis to yield **AM‐Rho**, showing increasing fluorescence at 565 nm (Figure 4b,c, red lines and dots). Notably, the fluorescence intensity ratio at 565–446 nm remained constant during irradiation, indicating that the generation of **AM‐Rho** and **COH** occurred synchronously, and that the released ONOO^−^ was efficiently captured by **CBE** in real‐time (Figure 4d). Subsequently, the ONOO^−^ release kinetics of photoactivated **O‐PND** were compared with that of the spontaneous donor SIN‐1, using **CBE** as the detection probe. The results showed that **O‐PND** completed ONOO^−^ release within 6 min, whereas SIN‐1 exhibited a much slower release profile, continuing to generate ONOO^−^ even after 40 min. Within the first 10 min, the amount of ONOO^−^ released by SIN‐1 was only about one‐10th that of **O‐PND** (Figure 4e). These findings underscored the suitability of **O‐PND** for applications requiring rapid and localized ONOO^−^ release and allows real‐time tracking of ONOO^−^ generation without auxiliary probes.

Similarly, the ONOO^−^ release from **Si‐PND** under light activation was assessed using **PY1**,^[15]^ a Rhodol‐based fluorescent probe that selectively responds to ONOO^−^ and emits in the green channel (Figure 4f). The fluorescence emission at 545 nm increased progressively with irradiation time (Figure 4g, red lines; Figure 4g, red dots), indicating efficient ONOO^−^ capture by **PY1**. Concurrently, the fluorescence of **Si‐Rho**, derived from **Si‐PND** photolysis, also intensified (Figure 4g blue lines, Figure 4h blue dots). The fluorescence intensity ratio between 647 and 545 nm remained constant, confirming the synchronous generation of ONOO^−^ and **Si‐Rho** (Figure 4i). The rate of ONOO^−^ release upon light activation of Si‐Rho is significantly faster than the spontaneous generation of ONOO^−^ by SIN‐1 (Figure 4j). These results demonstrated that **Si‐PND** also enables efficient and rapid ONOO^−^ release under light activation, accompanied by a built‐in self‐reporting mechanism.

**FIGURE 5:**
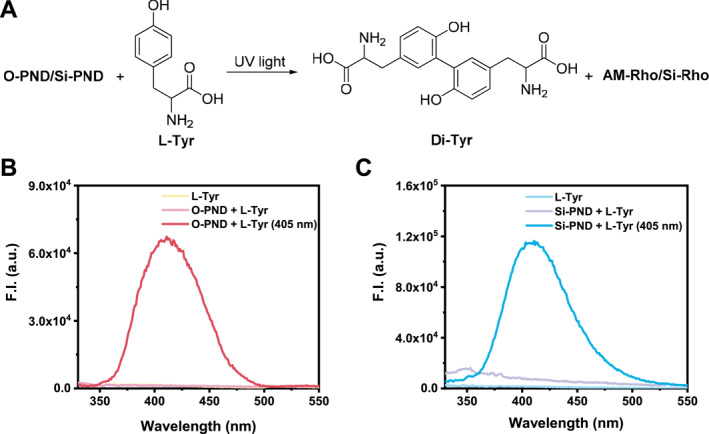
(a) Schematic illustration of photo‐triggered ONOO^−^ generation from **O‐PND** or **Si‐PND** leading to the formation of dityrosine (Di‐Tyr) from L‐tyrosine (L‐Tyr). Fluorescence emission spectral changes of mixtures containing L‐Tyr (0.5 mM) and (b) **O‐PND** (0.1 mM) or (c) **Si‐PND** (0.1 mM) in phosphate buffer (50 mM, pH 8.2) containing NaHCO_3_ (15 mM) before and after 405 nm light irradiation. *λ*_ex_ = 313 nm.

Peroxynitrite is an exceptionally potent agent with both nitrating and oxidizing properties. To further validate the photoinduced ONOO^−^ release from the donors, L‐tyrosine (L‐Tyr) was employed as a chemical probe. In a weakly alkaline phosphate buffer (pH 8.2) containing NaHCO_3_, ONOO^−^ oxidizes L‐Tyr to generate tyrosyl radicals, which subsequently undergo dimerization to form 3,3′‐dityrosine (Di‐Tyr).^[16]^ Di‐Tyr exhibits characteristic blue fluorescence upon excitation at 313 nm, with emission centered around 400 nm (Figure 5a). Thus, fluorescence detection of Di‐Tyr provides a reliable approach to monitor ONOO^−^ generation. As shown in the fluorescence spectra of the **O‐PND** and L‐Tyr mixture under 405 nm LED irradiation, negligible fluorescence was observed around 400 nm before irradiation (Figure 5b). Upon light exposure, a prominent fluorescence signal emerged near 400 nm with approximately 40‐fold increase in intensity, confirming the efficient generation of ONOO^−^ from **O‐PND** (Figure 5b). An analogous increase was observed in the **Si‐PND** and L‐Tyr system (Figure 5c), further supporting the photo‐triggered ONOO^−^ release capability of **Si‐PND**. Photo‐triggered ONOO^−^ generation from **O‐PND** or **Si‐PND** was further confirmed by their ability to nitrate tyrosine,^[17]^ as demonstrated by the reaction with Ac‐Tyr‐OEt, yielding Ac‐nitroTyr‐OEt, as confirmed by HRMS (Figure S1). The dual reactivity of ONOO^−^ generated upon **PND** photolysis, involving both oxidation and nitration of tyrosine, makes **PND** a powerful molecular tool for investigating the mechanisms of ONOO^−^‐induced cellular damage.

### 2.4 Intracellular photolysis of O‐PND and Si‐PND and their release of ONOO^−^

**FIGURE 6:**
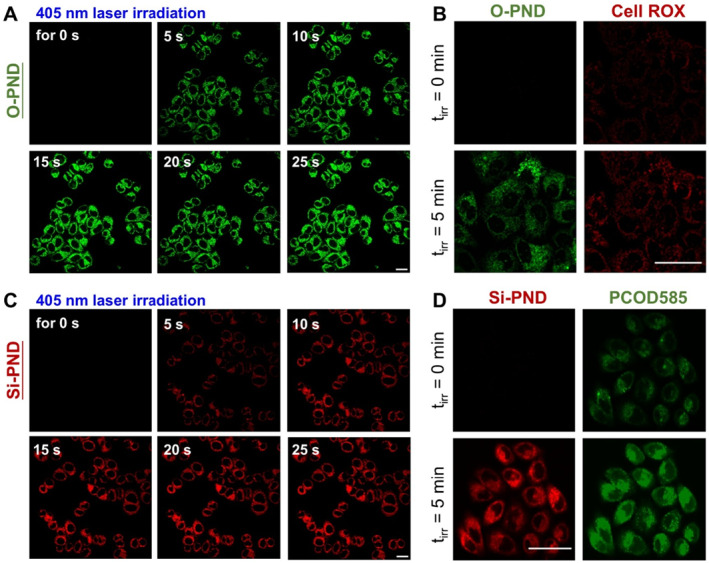
In vitro photoactivation of **O‐PND** and **Si‐PND**. (a) The confocal images of HeLa cells incubated with **O‐PND** (5 μM) upon irradiation by a built‐in 405 nm laser for the specified period. (b) The confocal images of HeLa cells incubated with **O‐PND** (5 μM) and CellROX^®^ Deep Red (10 μM) before and after irradiation with a 405 nm LED light (10 mW cm^−2^) for 5 min. (c) The confocal images of HeLa cells incubated with **Si‐PND** (5 μM) upon irradiation by a built‐in 405 nm laser for the specified period. (d) The confocal images of HeLa cells incubated with **Si‐PND** (5 μM) and **PCOD585** (5 μM) before and after irradiation with a 405 nm LED light (10 mW cm^−2^) for 5 min. Scale bar = 50 μm.

The intracellular behavior of **O‐PND** and **Si‐PND** was subsequently investigated. HeLa cells were incubated with either **O‐PND** (5 μM) or **Si‐PND** (5 μM), followed by irradiation with a built‐in 405 nm laser of the confocal microscope. Fluorescence images were acquired at 5‐s intervals until the fluorescence intensity reached a stable plateau. Prior to irradiation, no detectable fluorescence was observed in the cells, indicating that both **O‐PND** and **Si‐PND** remained in their non‐fluorescent inactive forms. This was consistent with the results of selectivity tests conducted at the solution level. Both **O‐PND** and **Si‐PND** exhibited high stability toward a broad range of biologically relevant species (Figure S2), ensuring minimal premature activation under physiological conditions. Upon 405 nm laser exposure for just 5 s, **O‐PND** rapidly produced a strong fluorescence signal in the rhodamine channel (*λ*_ex_: 561 nm, *λ*_em_: 570–620 nm), indicating its efficient photoactivation into a rhodamine fluorophore with ultrafast kinetics (Figure 6a). The fluorescence intensity gradually increased and plateaued at approximately 25 s, suggesting sustained formation of the photoactivatable dye and progressive depletion of **O‐PND** during the activation process. A similar fluorescence activation profile was observed for **Si‐PND** under identical conditions (Figure 6c), confirming efficient photo‐induced release of **Si‐Rho** in live cells. To verify ONOO^−^ release upon light activation, CellRox Deep Red^[18]^ and **PCOD585** ^[19]^ were used as ONOO^−^ ‐responsive fluorescent probes for **O‐PND** and **Si‐PND**, respectively. HeLa cells co‐incubated with **O‐PND** (5 μM) and CellRox Deep Red (5 μM) without light exposure showed weak fluorescence in both red and green channels. In contrast, cells irradiated with a 405 nm LED for 5 min exhibited markedly enhanced fluorescence signals (Figure 6b). The strong red fluorescence indicated that photogenerated ONOO^−^ activated CellRox Deep Red while the green fluorescence arose from the photoactivated rhodamine dye. Similarly, in cells co‐incubated with **Si‐PND** (5 μM) and **PCOD585** (5 μM), light irradiation triggered strong green fluorescence from **PCOD585**, confirming ONOO^−^ release, along with red fluorescence from the newly formed **Si‐Rho** (Figure 6d). These findings demonstrated that both **O‐PND** and **Si‐PND** were cell‐permeable, efficiently photoactivated, and effectively generated ONOO^−^ in situ, leading to elevated intracellular oxidative stress. Their efficient cell permeability, robust photoreactivity, effective ONOO^−^ generation, and high biological stability highlighted the potential of **O‐PND** and **Si‐PND** as reliable tools for precise oxidative stress modulation and related biomedical applications.

### 2.5 Intracellular burst of photo‐triggered ONOO^−^ in macrophages

**FIGURE 7:**
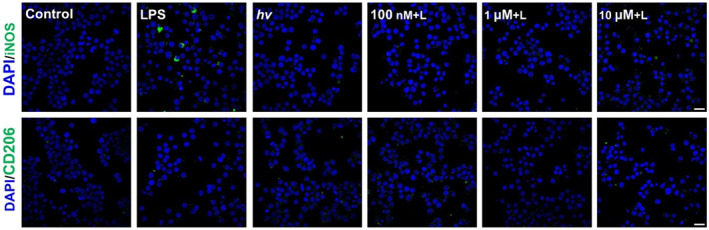
Immunofluorescence analysis of macrophage polarization in RAW264.7 cells induced by photo‐triggered ONOO^−^ release from **Si‐PND**. M1 marker iNOS and M2 marker CD206 are labeled in green, and cell nuclei are counterstained with DAPI (blue). Scale bar: 20 μm. “L” refers to irradiation with a 405 nm LED light (33 mW/cm^2^) for 5 min.

Macrophage polarization plays a critical role in regulating immune responses, with macrophages adopting either a pro‐inflammatory (M1) or anti‐inflammatory (M2) phenotype in response to the surrounding stimuli.^[20]^ While reactive oxygen species (ROS) play a complicated role in regulating macrophage polarization,^[21]^ the specific role of ONOO^−^ in this process remains poorly understood, largely due to the lack of effective strategies for its controlled generation in live cells. To address this gap, we evaluated the effect of photo‐triggered ONOO^−^ release from **Si‐PND** on macrophage polarization. It is well established that lipopolysaccharide (LPS)‐induced ROS generation promotes M1 polarization.^[22]^ Consistent with this, immunofluorescence staining revealed that LPS‐treated RAW264.7 cells exhibited increased expression of iNOS (a representative M1 marker) compared with untreated controls, while CD206 (an M2 marker) expression remained unchanged (Figure 7). In contrast, RAW264.7 cells treated with **Si‐PND** at varying concentrations (100 nM, 1 μM or 10 μM), followed by 405 nm light irradiation for 5 min, did not show increased iNOS expression, indicating that photo‐generated ONOO^−^ did not induce M1 polarization. Similarly, CD206 expression also remained unchanged, suggesting that ONOO^−^ release failed to promote M2 polarization. These results suggest that a transient burst of intracellular ONOO^−^ generated by photoactivation is insufficient to drive macrophage differentiation toward either phenotype, highlighting the role of ONOO^−^ as a modulator rather than a sole driver in macrophage polarization.

## 3 CONCLUSION

In summary, we have developed a novel class of photo‐triggered peroxynitrite (ONOO^−^) donors, **O‐PND** and **Si‐PND**, constructed from a single rhodamine‐based small‐molecule scaffold. These donors enabled precise ONOO^−^ release upon blue light irradiation and simultaneously furnished an intrinsic fluorescence readout, enabling real‐time visualization of the activation event. The released ONOO^−^ could be specifically detected by fluorescence probes and efficiently oxidizes and nitrates tyrosine. Both donors exhibited good stability toward biorelevant species, high cell permeability, and robust photo‐responsiveness, allowing reliable ONOO^−^ delivery under physiological environments. Cellular studies confirmed their ability to elevate intracellular oxidative stress upon light activation. However, the rapid and transient ONOO^−^ burst was insufficient to drive significant macrophage polarization, highlighting the complexity of redox signaling in immune regulation. Collectively, these well‐characterized molecular tools provide a powerful platform for spatiotemporally controlled ONOO^−^ delivery and offer valuable insights for further investigation of ONOO^−^‐mediated biological processes and potential therapeutic applications. Future studies will aim to elucidate downstream targets of donor‐derived ONOO^−^ signaling and validate donor performance in ONOO^−^‐related disease models, thereby enhancing their translational relevance. In parallel, we will explore red/NIR‐responsive analogs with longer excitation wavelengths to improve tissue penetration and expand in vivo applicability.

## CONFLICT OF INTEREST STATEMENT

The authors declare no conflicts of interest.

## ETHICS STATEMENT

No animal or human experiments were involved in this study.

## Supporting information

Supporting Information S1

## Data Availability

Supporting Information is available from the Wiley Online Library or from the author.
