# Supplementary material for: A single‐molecule platform for photo‐triggered peroxynitrite release with built‐in fluorescence calibration
Source: Smart Mol. 2025 Aug 14;3(3):e70013. doi: 10.1002/smo2.70013 (PMC12483127; doi:10.1002/smo2.70013)
Supplement: Supplementary file 1 — Supporting Information S1 [file SMO2-3-e70013-s001.docx]

Supporting Information

for

**A Single-Molecule Platform for Photo-Triggered Peroxynitrite Release with Built-in Fluorescence Calibration**

Mengqi Liu^#^, Yufeng Zhu^#^, Xiaoyue Zhang^#^, Yanyan Hou, Huan Zhang, Francisco Galindo, Youjun Yang*, Xuhong Qian*, Xiao Luo*

M. Liu; Y. Zhu; X. Zhang, Y. Hou; Y. Yang.

State Key Laboratory of Bioreactor Engineering, Shanghai Key Laboratory of Chemical Biology, School of Pharmacy, East China University of Science and Technology, Meilong Road 130, Shanghai 200237, China.
E-mail: youjunyang@ecust.edu.cn

X. Qian; X. Luo.
Shanghai Engineering Research Center of Molecular Therapeutics and New Drug Development, School of Chemistry and Molecular Engineering, East China Normal University, Shanghai 200241, China;

E-mail: xhqian@ecnu.edu.cn; xluo@chem.ecnu.edu.cn

Francisco Galindo

Departamento de Química Inorgánica y Orgánica, Universitat Jaume I de Castellón, Avda.Vicente Sos Baynat s/n, 12071 Castellón de la Plana, Spain.

Table of Contents

| **Contents** | Page |
| --- | --- |
| General methods | S3 |
| Experimental Section | S3-S5 |
| Figure S1. Photo-triggered ONOO^-^ generation from **O-PND** or **Si-PND** to react with Ac-Tyr-OEt, yielding Ac-nitroTyr-OEt. | S6 |
| Figure S2. Stability of **O-PND** (5 μM) and **Si-PND** (2 μM) toward various biologically relevant species. | S6 |
| Figure S3. Colocalization study of **O-PND** in HeLa cells. | S7 |
| Figure S4. The dark-toxicity and phototoxicity of **O-PND** towards Hela cells | S7 |
| **Figure S5.** Cell viability of RAW264.7 cells after treatment with **Si-PND** (5 μM) and exposure to 405 nm LED light (33 mW/cm²) for different durations. | S8 |
| **Figure S6.** Fluorescence intensity at 565 nm (for **O-PND**) or 647 nm (for **Si-PND**) as a function of irradiation time under continuous exposure to 405 nm LED light (100 mW/cm²). | S8 |
| **Figure S7**. Quantification of ONOO⁻ generation during the photolysis of **O-PND** and **Si-PND**. | S8 |
| **Figure S8**. EPR spectra of solutions containing PTIO (20 μM) and **O-PND** (20 μM) or **Si-PND** (20 μM) in PBS (50 mM, pH 7.4) with 5% DMF, recorded after irradiation with a 365 nm LED light (15 mW cm⁻²) for 10 min. | S9 |
| **Scheme S1**. Synthetic pathways of **O-PND**. | S10 |
| **Scheme S2**. Synthetic pathways of **Si-PND**. | S12 |
| **Figure S9-S30**. The ^1^H-NMR, ^13^C-NMR and HRMS spectra of all compounds. | S16-26 |
| References of supplementary information | S27 |

**General methods**

Unless otherwise stated, all chemicals were purchased from reputable commercial suppliers in China and used without further purification. Reaction progress was monitored by thin-layer chromatography (TLC). Column chromatography was performed using silica gel (300–400 mesh) from Qingdao Ocean Chemicals. The Cell Counting Kit-8 (CCK-8) was obtained from TargetMol. ^1^H-NMR and ^13^C-NMR spectra were recorded on Bruker AV-400 MHz or AV-600 MHz spectrometers, with chemical shifts reported in parts per million (ppm) relative to tetramethylsilane (TMS) or residual solvent peaks as internal standards. High-resolution mass spectrometry (HRMS) data were acquired using a Waters Xevo-G2 TOF mass spectrometer. UV-Vis absorption spectra were recorded using a SHIMADZU UV-2600 UV–vis spectrophotometer with a 1 cm quartz cuvette. Fluorescence spectra were obtained on a PTI-QM4 steady-stead fluorimeter equipped with a 75 W Xenon arc-lamp and a model 810 PMT (voltage 950 V). The excitation and emission slit widths were set to 2 nm, with an integration time of 0.1 s. Confocal fluorescence imaging was performed on an Olympus FV3000 confocal microscope, and bright-field images were captured using an OPTIKA IM-3 microscope. Photolysis experiments were conducted using a Photosyn-10 parallel photoreactor (Shanghai Quanhuan Technology Co., Ltd.).

**Experimental Section**

***Photoactivation of O-PND and Si-PND in cuvettes.***

A **PND** (5 μM) solution in PBS (10 mM, pH = 7.4) containing 1% DMSO was irradiated by 365 nm, 405 nm, 440 nm LED lights at different power intensities for different time durations. The UV-Vis absorption and fluorescence spectra were acquired immediately.

***Detection of ONOO^-^ with CBE or PY1***

A solution of **O-PND** (5 μM) and **CBE** (5 μM) was prepared in phosphate-buffered saline (PBS, pH 7.4) containing 1% DMSO. The mixture was irradiated with a 405 nm LED light for different time intervals. Fluorescence emission spectra were recorded using a spectrofluorometer by excitation at 325 nm for the ONOO⁻-responsive coumarin emission (**COH**) and at 540 nm for the internal fluorophore **AM-Rho**.

A solution of **Si-PND** (5 μM) and **PY1** (5 μM) was prepared in phosphate-buffered saline (PBS, pH 7.4) containing 1% DMSO. The mixture was irradiated with a 405 nm LED light for different time intervals. Fluorescence emission spectra were recorded using a spectrofluorometer by excitation at 545 nm for the ONOO⁻-responsive **Rhodol** emission and at 647 nm for the internal fluorophore **Si-Rho**.

***Detection of ONOO^-^ with L-Tyr***

Mixtures containing **O-PND** (0.1 mM) or **Si-PND** (0.1 mM) and L-Tyr (0.5 mM) were prepared in phosphate buffer (50 mM, pH 8.2) containing NaHCO_3_ (15 mM) to mimic a CO₂-rich physiological environment. The solutions were irradiated with a 405 nm LED light (20 mW cm^-2^) for 5 min. The fluorescence emission spectra were recorded using a spectrofluorometer with an excitation wavelength of 313 nm, and the emission was monitored from 350 to 500 nm.

***Photolysis monitoring of PND by HPLC analysis***

A 0.5 mM solution of PND in PBS with 5% MeCN was prepared and irradiated with a 365 nm LED (100 mW/cm²) for varying periods of time. The resulting solution at different intervals was analyzed by HPLC. HPLC analysis of the **PND** photolysis was performed using acetonitrile and water (containing 0.1% trifluoroacetic acid) as eluents. A flow rate of 1 mL/min was applied with a gradient elution program (0-8 min, 30%-95% acetonitrile; 8-20 min, 95% acetonitrile). The detection wavelength was set to 254 nm.

***NO Trapping with PTIO***

2-Phenyl-4,4,5,5-tetramethylimidazoline-1-oxyl-3-oxide (PTIO) was used as a spin-trapping agent for NO. Aqueous solutions (with 5% DMF) containing PTIO (20 μM) and either O-PND (20 μM) or Si-PND (20 μM) were irradiated with 365 nm LED light (15 mW/cm²) for 10 minutes. Electron paramagnetic resonance (EPR) spectra were then recorded using a Bruker EMXPLUS-10/12 spectrometer (Bruker Analytische Messtechnik GmbH, Germany).

***Cell culture***

Hela cells were cultured in DMEM medium supplemented with 10% fetal bovine serum (FBS) and 1% penicillin-streptomycin. The cells were maintained in a humidified incubator at 37°C with 5% CO₂.

***Colocalization study***

HeLa cells were seeded at a density of 1×10^5^ cells per 35 mm glass-bottomed culture dish. After cell adhesion, the cells were incubated with **O-PND** (2.5 μM) for 30 min and washed three times with PBS to remove unbound molecules. Subsequently, commercial markers for mitochondria, endoplasmic reticulum, and lysosomes were used sequentially for staining (following the manufacturer’s instructions): each dye was incubated for 30 min, followed by three washes with PBS to eliminate excess dye. Prior to imaging with a confocal fluorescence microscope (Olympus FV 3000), the treated HeLa cells were irradiated with the built-in 405 nm laser for 5 seconds. Fluorescence colocalization images were analyzed quantitatively using ImageJ. Mito Tracker™ Deep Red FM ( Thermo Fisher Scientific): λ_ex_=640 nm，λ_em_= 650-750 nm; ER-Tracker™ Blue-White DPX (Thermo Fisher Scientific): λ_ex_=374 nm, λ_em_= 430-470 nm, Lysosome Probe Deep Red (BioLegend): λ_ex_=596 nm, λ_em_= 610-650 nm.

***Intracellular detection of ONOO^-^ with CellRox Deep Red and PCOD585***

Hela cells were seeded in a 35 mm glass-bottom culture dish. In a dark environment, the cells were treated with **O-PND** or **Si-PND** (5 μM) for 45 minutes, followed by the addition of CellRox Deep Red (5 μM) or **PCOD585** (5 μM) for an additional 30-minute incubation and washed three times with PBS. The experimental and control groups were exposed to a 405 nm laser or kept in the dark, respectively, prior to capturing the fluorescence images.

***Immunofluorescence stain of macrophage***

Immunofluorescence staining was performed to detect macrophage polarization markers iNOS and CD206. RAW264.7 cells (6×10^5^ cells/well) were seeded in 35 mm glass-bottom dishes, medium containing different concentrations of **Si-PND** for 1 h, and irradiated with 405 nm LED for 5 min. After 24 h culture, cells were fixed with 4% paraformaldehyde for 15 min, permeabilized, and blocked, then incubated overnight at 4°C with anti-iNOS (Huabio) or anti-CD206 (ProteinTech) primary antibodies. The next day, cells were incubated with CoraLite® Plus 488-conjugated secondary antibody for 1 h, stained with DAPI (PBS-diluted) for 5 min, and observed by fluorescence confocal microscopy.

**Figure S1**. Photo-triggered ONOO^-^ generation from **O-PND** or **Si-PND** to react with Ac-Tyr-OEt, yielding Ac-nitroTyr-OEt.

**Figure S2.** Stability of **O-PND** (5 μM) and **Si-PND** (2 μM) toward various biologically relevant species (100 μM Cys, GSH, Vc, ClO^−^, ONOO^−^, ·OH, ^1^O_2_, O_2_^•−^, H_2_O_2_) in phosphate buffer saline (50 mM at pH = 7.4) with 1% DMSO as a co-solvent.

**Figure S3**. Colocalization study of **O-PND** in HeLa cells with commercial Lyso-, Mito- or ER Tracker. Scale bar, 50 μm.

**Figure S4**. The dark-toxicity and phototoxicity of **O-PND** towards Hela cells (405 nm, 33 mW/cm^2^, irradiation for 6 min).

**Figure S5.** Cell viability of RAW264.7 cells after treatment with **Si-PND** (5 μM) and exposure to 405 nm LED light (33 mW/cm²) for different durations.

**Figure S6.** Fluorescence intensity at 565 nm (for **O-PND**) or 647 nm (for **Si-PND**) as a function of irradiation time under continuous exposure to 405 nm LED light (100 mW/cm²).

**Figure S7**. Quantification of ONOO⁻ generation during the photolysis of **O-PND** and **Si-PND**. (A) Calibration curve of CBE fluorescence intensity at 446 nm versus ONOO⁻ concentration in PBS (50 mM, pH = 7.4). (B) Fluorescence spectra of CBE (5 μM) in the presence of **O-PND** or **Si-PND** (5 μM) upon irradiation with 365 nm LED light (30 mW cm⁻²) for 5 min in PBS (50 mM, pH 7.4) containing 5% DMF. The ONOO⁻ concentration was calculated based on the fluorescence intensity using the standard curve.

**Figure S8**. EPR spectra of solutions containing PTIO (20 μM) and **O-PND** (20 μM) or **Si-PND** (20 μM) in PBS (50 mM, pH 7.4) with 5% DMF, recorded after irradiation with a 365 nm LED light (15 mW cm⁻²) for 10 min.

**Scheme S1.** Synthetic pathway of **O-PND**.

***Synthesis of compound S2***

To a stirred solution of rhodamine 6G (**S1**, 20.0 g, 46.56 mmol, 1.0 eq.) in a mixture of 40 mL ethanol and 80 mL water was added NaOH (16.6 g, 46.56 mmol, 1.0 eq.) incrementally in several batches. The reaction mixture was then stirred and heated at 100 °C for 4 hours. Upon termination of the reaction, the reaction solution was poured onto a mass of ice. Under ice-bath conditions, the pH value was adjusted to 5-6 using concentrated hydrochloric acid to precipitate a red solid. The solid was suction-filtered, and the filter cake was washed three times with purified water and then dried, affording 15.4 g of the red solid of compound **S2**^1^ with a yield of 80%. ^1^H NMR (400 MHz, CD_3_OD) δ 8.32 (d, *J* = 7.5 Hz, 1H), 7.85 – 7.76 (m, 2H), 7.37 (d, *J* = 7.2 Hz, 1H), 6.90 (s, 4H), 3.51 (q, *J* = 7.2 Hz, 4H), 2.13 (s, 6H), 1.36 (t, *J* = 7.2 Hz, 6H).

***Synthesis of compound S3***

To a stirred solution of compound **S2** (10.0 g, 24.13 mmol, 1.0 eq.) in 20 mL water was added NaOH (34.0 g, 844.38 mmol, 35 eq.) incrementally in several batches. The reaction mixture was stirred at 100 °C for 5 hours. After the reaction was completed, the reaction solution was poured onto ice. Under ice-bath conditions, the pH was adjusted to 5-6 with concentrated hydrochloric acid to precipitate a yellow solid. The solid was suction-filtered, and the filter cake was washed three times with purified water and then dried, affording 5.5 g of the red solid of compound **S3**^2^ with a yield of 76% yield. ^1^H NMR (600 MHz, CDCl_3_) δ 12.59 (s, 1H), 8.11 (dd, *J* = 7.8, 0.8 Hz, 1H), 7.64 (td, *J* = 7.5, 1.2 Hz, 1H), 7.55 (td, *J* = 7.7, 1.2 Hz, 1H), 7.36 (dd, *J* = 7.5, 0.8 Hz, 1H), 6.67 (s, 1H), 6.13 (s, 1H), 3.26 (q, *J* = 7.2 Hz, 2H), 1.90 (s, 3H), 1.31 (t, *J* = 7.2 Hz, 3H).

***Synthesis of compound S4***

To a stirred solution of compound **S3** (5.0 g, 16.70 mmol, 1.0 eq.) in concentrated sulfuric acid (10 mL) was added 3-(diethylamino)phenol (3.3 g, 20.00 mmol, 1.2 equiv.). The reaction was heated at 120 ℃ overnight. After the reaction was completed, under ice-bath conditions, 6 M sodium hydroxide was added to adjust the pH to 5-6, resulting in the precipitation of a red solid. The solid was suction filtered, and the filter cake was washed three times with purified water and then dried. The crude product was purified by column chromatography (DCM:MeOH=20:1, v/v) to obtain the compound **S4**^3^ (5.4 g) as a red solid in a 75% yield. ^1^H NMR (400 MHz, CDCl_3_) δ 8.33 (d, *J* = 8.1 Hz, 1H), 7.69 – 7.61 (m, 2H), 7.15 (d, *J* = 6.8 Hz, 1H), 6.98 (d, *J* = 9.2 Hz, 1H), 6.72 – 6.62 (m, 3H), 6.47 (s, 1H), 3.52 (q, *J* = 6.9 Hz, 4H), 3.37 (ddd, *J* = 20.9, 13.4, 7.1 Hz, 2H), 1.99 (s, 3H), 1.27 (t, *J* = 7.0 Hz, 6H), 1.16 (t, *J* = 7.0 Hz, 3H).

***Synthesis of compound AM-Rho***

To a stirred solution of compound **S4** (3.0 g, 7.00 mmol, 1.0 eq.) in dry DMF (20 mL) was added N-ethyl diisopropylamine (5.5 mL, 28.70 mmol, 4.1 eq). After stirring for 2 minutes, bromomethyl acetate (2.5 mL, 25.20 mmol, 3.6 eq.) was added, and the mixture was stirred overnight at room temperature. After the reaction was completed, the reaction mixture was extracted with DCM repeatedly. The organic layer was dried with anhydrous Na_2_SO_4_, filtered, and concentrated under reduced pressure. The crude product was purified by column chromatography (DCM:MeOH=30:1, v/v) to obtain the compound **AM-Rho** (2.1 g) as a red solid in a 60% yield. ^1^H NMR (400 MHz, CDCl_3_) δ 8.61 (s, 1H), 8.30 (d, *J* = 7.8 Hz, 1H), 7.81 (t, *J* = 7.4 Hz, 1H), 7.73 (t, *J* = 7.6 Hz, 1H), 7.27 (d, *J* = 7.9 Hz, 1H), 6.95 (d, *J* = 9.3 Hz, 1H), 6.81 – 6.74 (m, 1H), 6.73 (s, 1H), 6.65 (s, 2H), 5.61 (q, *J* = 5.7 Hz, 2H), 3.73 – 3.59 (m, 2H), 3.55 (q, *J* = 6.9 Hz, 4H), 2.35 (s, 3H), 1.88 (s, 3H), 1.38 (t, *J* = 7.1 Hz, 3H), 1.27 (t, *J* = 7.0 Hz, 6H). ^13^C NMR (101 MHz, CDCl_3_) δ 169.15, 163.66, 158.01, 157.43, 156.93, 156.15, 154.24, 134.30, 133.66, 131.73, 130.38, 130.23, 128.63, 128.38, 127.94, 113.82, 113.13, 112.67, 96.04, 93.65, 79.40, 45.71, 38.52, 20.44, 19.30, 13.78, 12.52. ESI-HRMS, m/z, [M]^+^, calcd. for C_30_H_33_N_2_O_5_^+^, 501.2384; found 501.2390.

***Synthesis of compound 1***

To a stirred solution of compound **AM-Rho** (300.0 mg, 0.60 mmol, 1.0 equiv.) in acetic acid (20 mL) was added zinc powder (170.0 mg, 3.00 mmol, 5 eq.) and trifluoroacetic acid (500 μL) as a catalyst. The reaction was carried out overnight at room temperature. After the reaction was completed, the zinc powder was removed by suction filtration. The filtrate was collected and extracted with ethyl acetate repeatedly. The organic layer was dried with anhydrous Na_2_SO_4_, filtered, and concentrated under reduced pressure to obtain compound **1** (285.0 mg) as colorless oily liquid. This colorless oily liquid was used without further purification.

***Synthesis of O-PND***

A solution of compound **1** (285.0 mg, 0.57 mmol, 1.0 eq.) in dry THF (20 mL) was cooled to 0 ℃ under nitrogen, and a aqueous solution of sodium nitrite (2.1 mL, 0.85 mmol, 0.4 M in water. 1.5 eq.) was added and stirred for 20 minutes. Then, acetic acid (6 mL) was added and the reaction was continued for 1 hour. After the reaction was completed, the reaction mixture was extracted with ethyl acetate repeatedly. The organic layer was dried with anhydrous Na_2_SO_4_, filtered, and concentrated under reduced pressure. The crude product was purified by column chromatography (PE:EA = 15:1, v/v) to obtain the **O-PND** (181 mg) as a pink solid with a total yield of 60% over two steps. ^1^H NMR (400 MHz, CDCl_3_) δ 7.88 (d, *J* = 7.6 Hz, 1H), 7.39 (t, *J* = 7.5 Hz, 1H), 7.20 (dt, *J* = 20.7, 7.7 Hz, 2H), 7.04 (s, 1H), 6.99 (s, 1H), 6.95 (s, 1H), 6.82 (d, *J* = 8.6 Hz, 1H), 6.78 (d, *J* = 8.7 Hz, 1H), 6.69 (s, 1H), 6.38 (s, 1H), 6.34 (s, 1H), 6.30 (s, 1H), 6.09 – 6.02 (m, 2H), 4.54 – 4.42 (m, 1H), 3.99 – 3.89 (m, 1H), 3.33 (dd, *J* = 13.0, 6.1 Hz, 4H), 2.17 (s, 2H), 2.16 (s, 1H), 2.07 (s, 2H), 1.84 (s, 1H), 1.14 (dt, *J* = 17.7, 7.1 Hz, 8H). ^13^C NMR (101 MHz, CDCl_3_) δ 169.81, 166.81, 151.66, 149.91, 149.13, 148.05, 139.22, 133.51, 132.61, 132.22, 130.42, 130.07, 128.92, 127.81, 126.41, 126.35, 114.72, 110.89, 108.14, 98.61, 80.03, 44.50, 41.97, 37.72, 20.90, 17.56, 12.72, 11.40. ESI-HRMS, m/z, [M+H]^+^, calcd. for C_30_H_34_N_3_O_6_^+^, 532.2442; found 532.2449.

**Scheme S2.** Synthetic pathway of **Si-PND**.

***Synthesis of compound S6***

To a solution of compound **S5** (20.0 g, 116.27 mmol, 1.0 eq.) and paraformaldehyde (14.8 g, 523.25 mmol, 4.5 eq.) in methanol (100 mL) was added sodium methoxide (28.2 g, 581.35 mmol, 5.0 eq.). The mixture was stirred overnight at room temperature. After the reaction was completed, the reaction solution was cooled to 0 °C, and NaBH_4_ (6.0 g, 174.40 mmol, 1.5 eq.) was added in portions. Next, the reaction mixture was heated to 80 °C and allowed to react for another 2 hours. After cooling to room temperature, the reaction was quenched with 2 M sodium hydroxide solution and extracted with ethyl acetate repeatedly. The organic layer was dried with anhydrous Na_2_SO_4_, filtered, and concentrated under reduced pressure. The crude product was purified by column chromatography (PE:EA = 400:1, v/v) to obtain the compound **S6**^4^ (16.8 g) as yellow oily liquid in a 77% yield.

^1^H NMR (400 MHz, CDCl_3_) δ 7.05 (t, *J* = 8.0 Hz, 1H), 6.91 – 6.79 (m, 1H), 6.75 (t, *J* = 1.9 Hz, 1H), 6.52 (dd, *J* = 8.0, 1.9 Hz, 1H), 3.69 (s, 1H), 2.81 (s, 3H).

***Synthesis of compound* S7**

To a solution of compound **S6** (10.0 g, 53.75 mmol, 1.0 eq.) and K_2_CO_3_ (18.5 g, 134.37 mmol, 2.5 eq.) in MeCN (70 mL) was added allyl bromide (17.5 g, 145.12 mmol, 2.7 eq.). The mixture was stirred and reacted at 80 °C for 4 h. After the reaction was complete, it was cooled to room temperature. Filtration was carried out, and the filter cake was washed with acetonitrile three times. The filtrate was collected and distilled under reduced pressure. The crude product was purified by column chromatography (PE:EA = 200:1, v/v) to obtain the compound **S7**^5^ (10.9 g) as yellow liquid in a 90% yield.

^1^H NMR (400 MHz, CDCl_3_) δ 7.07 (t, *J* = 8.1 Hz, 1H), 6.84 (d, *J* = 2.2 Hz, 1H), 6.82 (d, *J* = 7.8 Hz, 1H), 6.63 (dd, *J* = 8.4, 2.1 Hz, 1H), 5.83 (ddt, *J* = 16.9, 10.1, 4.9 Hz, 1H), 5.21 – 5.17 (m, 1H), 5.15 (dd, *J* = 12.4, 1.6 Hz, 1H), 3.91 (d, *J* = 4.9 Hz, 2H), 2.94 (s, 3H).

***Synthesis of compound* S9**

To a mixture of compound **S8** (20.0 g, 98.52 mmol, 1.0 eq.) and K_2_CO_3_ (40.7 g, 295.56 mmol, 3.0 eq.) was added a 40% aqueous solution of dimethylamine (100 mL, 1.50 mol, 15.0 eq.), which served as both the solvent and the reactant. The reaction mixture was heated to 80 °C and allowed to react for 6 hours. After the reaction was completed, K_2_CO_3_ was removed by hot filtration. The filtrate was distilled under reduced pressure, and then ethanol was added. The solid was completely dissolve at 70 °C, and then the solution was quickly transferred to an ice-water bath, at which point yellow crystals precipitated. The yellow crystals were dried to obtain compound **S9**^6^ (18.6 g) in an 83% yield.

^1^H NMR (400 MHz, CDCl_3_) δ 10.06 (s, 1H), 7.78 (d, *J* = 8.9 Hz, 1H), 6.78 (d, *J* = 2.4 Hz, 1H), 6.61 (dd, *J* = 8.9, 2.4 Hz, 1H), 3.06 (s, 6H).

***Synthesis of compound S10***

To a solution of compound **S9** (10.0 g, 44.24 mmol, 1.0 eq.) in a mixture of 48 mL dichloromethane and 12 mL methanol was cooled to 0 ℃ under nitrogen, and NaBH_4_ (2.5 g, 66.37 mmol, 1.5 eq.) was added in small portions several times. The reaction mixture was stirred and allowed to react for 0.5 h. After the reaction was completed, the reaction was quenched with NH_4_Cl solution and extracted with CH_2_Cl_2_ repeatedly.

The organic layer was dried with anhydrous Na_2_SO_4_, filtered, and concentrated under reduced pressure, and crude product of compound **S10**^7^ (9.6 g) was acquired as a white solid with a yield of 95%.

^1^H NMR (400 MHz, CDCl_3_) δ 7.24 (d, *J* = 8.4 Hz, 1H), 6.90 (d, *J* = 2.6 Hz,1H), 6.65 (dd, *J* = 8.4 Hz, 2.6 Hz, 1H), 4.64 (s, 2H), 2.94 (s, 6H).

***Synthesis of compound S11***

To a solution of compound **S7** (9.7 g, 42.90 mmol, 1.0 eq.) and compound ***S10*** (9.8 g, 42.90 mmol, 1.0 eq.) in dichloromethane (50 mL) was added BF_3_·OEt_2_ (8 mL, 64.35 mmol, 1.5 eq.) dropwise under ice-bath conditions. Then, the mixture was stirred overnight at room temperature. After the reaction was completed, the reaction was quenched with H_2_O solution and extracted with CH_2_Cl_2_ repeatedly. The organic layer was dried with anhydrous Na_2_SO_4_, filtered, and concentrated under reduced pressure. The crude product was purified by column chromatography (PE:EA = 300:1, v/v) to obtain the compound **S11**^5^ (8.9 g) as a white solid in a 48% yield.

^1^H NMR (600 MHz, CDCl_3_) δ 6.94 (dd, *J* = 9.6, 2.6 Hz, 2H), 6.85 (dd, *J* = 18.0, 8.6 Hz, 2H), 6.59 (ddd, *J* = 13.9, 8.6, 2.6 Hz, 2H), 5.82 (ddt, *J* = 17.1, 9.9, 5.0 Hz, 1H), 5.19 – 5.14 (m, 2H), 4.00 (s, 2H), 3.88 (d, *J* = 5.0 Hz, 2H), 2.92 (s, 6H), 2.91 (s, 3H).

***Synthesis of compound S13***

In a dry Schlenk flask, compound **S11** (6.0 g, 13.69 mmol, 1.0 eq.) was dissolved in dry THF (30 mL). After the gas was exchanged three times, an argon balloon was connected as a buffer. The reaction flask was placed in an environment at -78 °C and stirred for 20 minutes. Then, n-butyllithium (13.7 mL, 34.40 mmol, 2.5 M in hexanes, 2.4 eq.) was added dropwise, and the reaction was continued with stirring for 0.5 h at -78 ℃. Next, a solution of dichlorodimethylsilane (3.2 mL, 34.40 mmol, 2.4 eq.) was added, and the reaction was stirred for 10 minutes, the reaction system was transferred to room temperature and allowed to react for another 2 h. The reaction was quenched with 2 M hydrochloric acid solution and then distilled under reduced pressure until dry to obtain the crude product of compound **2** as a pale blue solid. The crude product was dissolved in dichloromethane (30 mL). At 0 °C, 2,3-dichloro-5,6-dicyano-1,4-benzoquinone (DDQ) (2.4 g, 13.76 mmol, 1.0 eq.) was added in batches. Then, the reaction system was transferred to room temperature and stirred for 2 h. After that, it was distilled under reduced pressure. Subsequently, DMF (50 mL) was added as a solvent, along with NaOH (1.1 g, 27.52 mmol, 2.0 eq.) and H_2_O (2.5 mL). After the reaction was completed, the excess iodine was quenched by adding sodium thiosulfate solution and extracted with CH_2_Cl_2_ repeatedly. The organic layer was dried with anhydrous Na_2_SO_4_, filtered, and concentrated under reduced pressure. The crude product was purified by column chromatography (PE:EA = 40:1, v/v) to obtain the compound **S13**^5^ (1.2 g) as a yellow solid in a 24% yield.

^1^H NMR (400 MHz, CDCl_3_) δ 8.39 (t, *J* = 8.5 Hz, 2H), 6.91 – 6.77 (m, 4H), 5.87 (ddd, *J* = 15.5, 10.1, 4.9 Hz, 1H), 5.21 (d, *J* = 2.8 Hz, 1H), 5.18 (d, *J* = 11.1 Hz, 1H), 4.04 (d, *J* = 4.8 Hz, 2H), 3.09 (d, *J* = 6.4 Hz, 9H), 0.46 (s, 6H).

***Synthesis of compound S14***

To a solution of compound **S13** (1.0 g, 2.85 mmol, 1.0 eq.) in dry DCM (20 mL) was added N,N-dimethylbarbituric acid (NDMBA) (668.0 mg, 4.28 mmol, 1.5 eq.). The mixture was stirred and purged with argon for 15 min before Pd(pph_3_)_4_ (330.0 mg, 0.28 mmol, 0.1 eq.) was added. The reaction was purged with argon for another 15 min. Then, the reaction was stirred overnight at 40 °C before cooled to room temperature and it was subsequently neutralized with saturated NaHCO_3_ solution. Then, the reaction mixture was extracted with CH_2_Cl_2_ repeatedly. The organic layer was dried with anhydrous Na_2_SO_4_, filtered, and concentrated under reduced pressure. The crude product was purified by column chromatography (PE:EA = 25:1, v/v) to obtain the compound **S14**^5^ (665 mg) as a yellow solid in a 75% yield.

^1^H NMR (400 MHz, CDCl_3_) δ 8.41 – 8.35 (m, 2H), 6.85 (dd, *J* = 9.0, 2.7 Hz, 1H), 6.81 (d, *J* = 2.6 Hz, 1H), 6.74 (d, *J* = 2.6 Hz, 1H), 6.72 (s, 1H), 3.10 (s, 6H), 2.94 (s, 3H), 0.45 (s, 6H).

***Synthesis of compound Si-Rho***

To a stirred solution of compound **S14** (600 mg, 2.13 mmol, 1.0 eq.) in dry THF (30 mL) was stirred under nitrogen. o-tolylmagnesium bromide (37.0 mL, 213.0 mmol, 100.0 eq.) was added dropwise at 0 ^o^C. Then, the reaction was heated at 70 ^o^C under argon for 6 h. It was subsequently quenched with 1 M hydrochloric acid solution and extracted with CH_2_Cl_2_ repeatedly. The organic layer dried with anhydrous Na_2_SO_4_, filtered, and concentrated under reduced pressure. The crude product was purified by column chromatography (DCM:MeOH = 30:1, v/v) to obtain the compound **Si-Rho** (445 mg) as a blue solid in a 60% yield.

^1^H NMR (600 MHz, CDCl_3_) δ 10.14 (s, 1H), 7.79 (s, 1H), 7.38 (t, *J* = 7.4 Hz, 1H), 7.28 (dd, *J* = 12.3, 7.4 Hz, 2H), 7.12 – 6.98 (m, 3H), 6.96 (d, *J* = 9.4 Hz, 1H), 6.51 (dd, *J* = 9.5, 2.7 Hz, 1H), 6.41 (s, 1H), 3.23 (s, 6H), 3.07 (s, 3H), 1.99 (s, 3H), 0.54 (s, 3H), 0.52 (s, 3H). ^13^C NMR (151 MHz, CDCl_3_) δ 168.52, 156.62, 152.91, 147.40, 146.68, 144.37, 139.95, 138.70, 135.75, 130.22, 128.98, 128.77, 127.72, 127.40, 127.06, 125.62, 119.26, 113.15, 112.03, 40.56, 29.82, 29.72, 19.43, -0.82, -1.14. ESI-HRMS, m/z, [M]^+^, calcd. for C_25_H_29_N_2_Si^+^, 385.2095; found 385.2099.

***Synthesis of compound 2***

To a solution of compound **Si-Rho** (300.0 mg, 0.78 mmol, 1.0 eq.) in methanol (30 mL) was added NaBH_4_ (44.2 mg, 1.17 mmol, 1.5 eq.) in small portions for several times. The reaction mixture was stirred at room temperature for 3 hours. After the reaction was completed, the reaction was quenched with NH_4_Cl solution and extracted with CH_2_Cl_2_ repeatedly. The organic layer was dried with anhydrous Na_2_SO_4_, filtered, and concentrated under reduced pressure, and crude product of compound **2** (293 mg) was acquired as a white solid and was directly used in the next step without further purification.

***Synthesis of Si-PND***

To a solution of compound **2** (293.0 mg, 0.76 mmol, 1.0 eq.) in dry THF (30 mL) was cooled to 0 ℃ under nitrogen, and a solution of sodium nitrite aqueous (1.9 mL, 0.76 mmol, 0.4 M in water. 1.0 eq.) was added and stirred for 20 minutes. Then, acetic acid (6 mL) was added and the reaction was continued for 3 hours. After the reaction was completed, it was subsequently neutralized with saturated NaHCO_3_ solution and extracted with ethyl acetate repeatedly. The organic layer was dried with anhydrous Na_2_SO_4_, filtered, and concentrated under reduced pressure. The crude product was purified by column chromatography (PE:EA = 10:1, v/v) to obtain the **Si-PND** (99 mg) as a yellow solid in a 31% yield over two steps.

^1^H NMR (400 MHz, CDCl_3_) δ 7.78 (d, *J* = 2.6 Hz, 1H), 7.32 (dd, *J* = 8.6, 2.6 Hz, 1H), 7.20 – 7.07 (m, 5H), 6.93 (dd, *J* = 12.1, 5.9 Hz, 2H), 6.69 (dd, *J* = 8.8, 2.9 Hz, 1H), 5.67 (s, 1H), 3.46 (s, 3H), 2.95 (s, 6H), 2.27 (s, 3H), 0.65 (s, 3H), 0.47 (s, 3H). ^13^C NMR (101 MHz, CDCl_3_) δ 148.37, 148.15, 144.95, 139.57, 135.98, 135.73, 135.49, 132.91, 131.34, 131.16, 130.18, 129.90, 126.32, 123.37, 120.00, 116.13, 114.81, 50.39, 40.58, 31.55, 30.16, 20.56, -0.35, -1.03. ESI-HRMS, m/z, [M+H]^+^, calcd. for C_25_H_30_N_3_OSi^+^, 416.2153; found 416.2160.


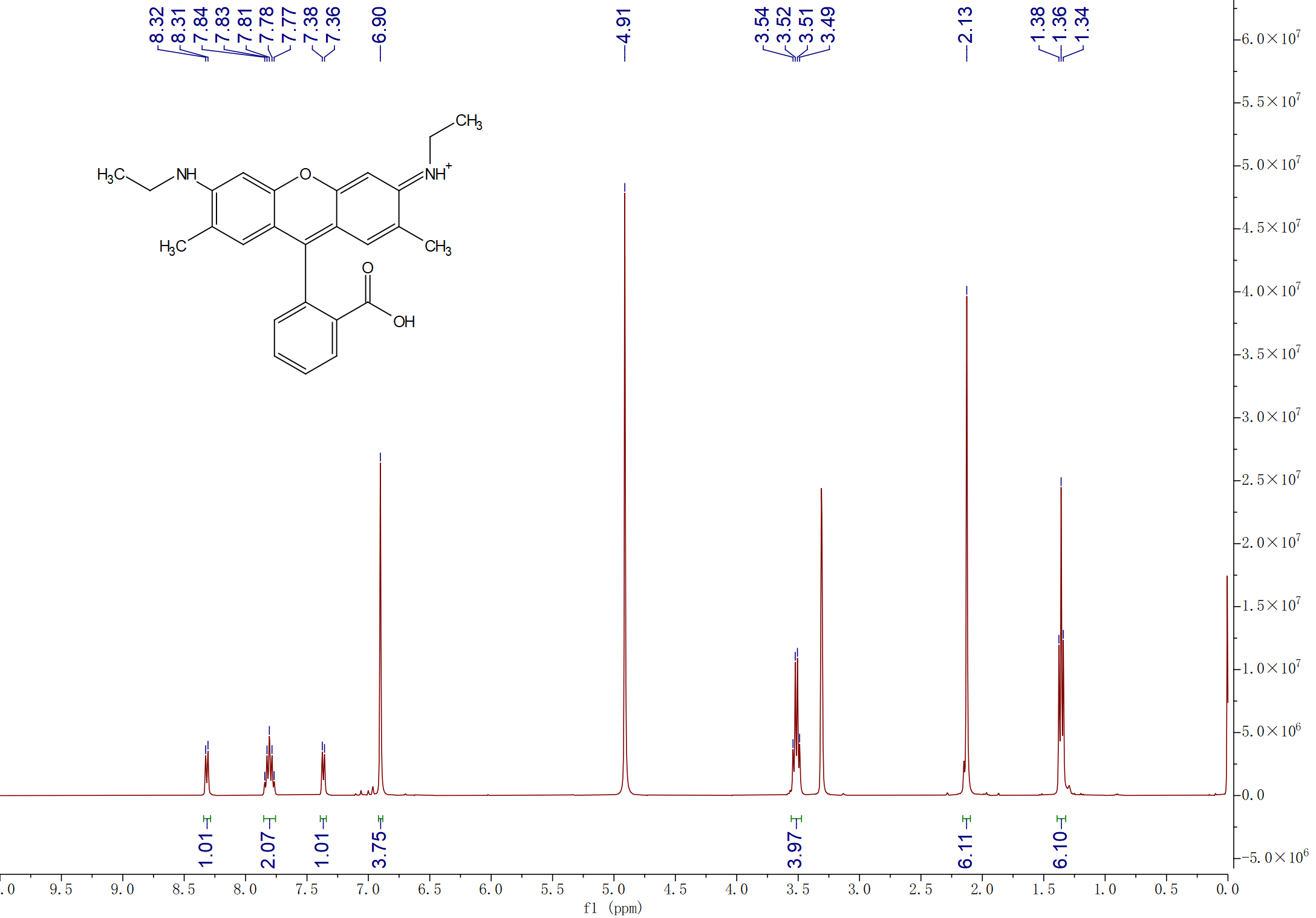


**Figure S9**. ^1^H-NMR spectrum of compound **S2** in CD_3_OD.


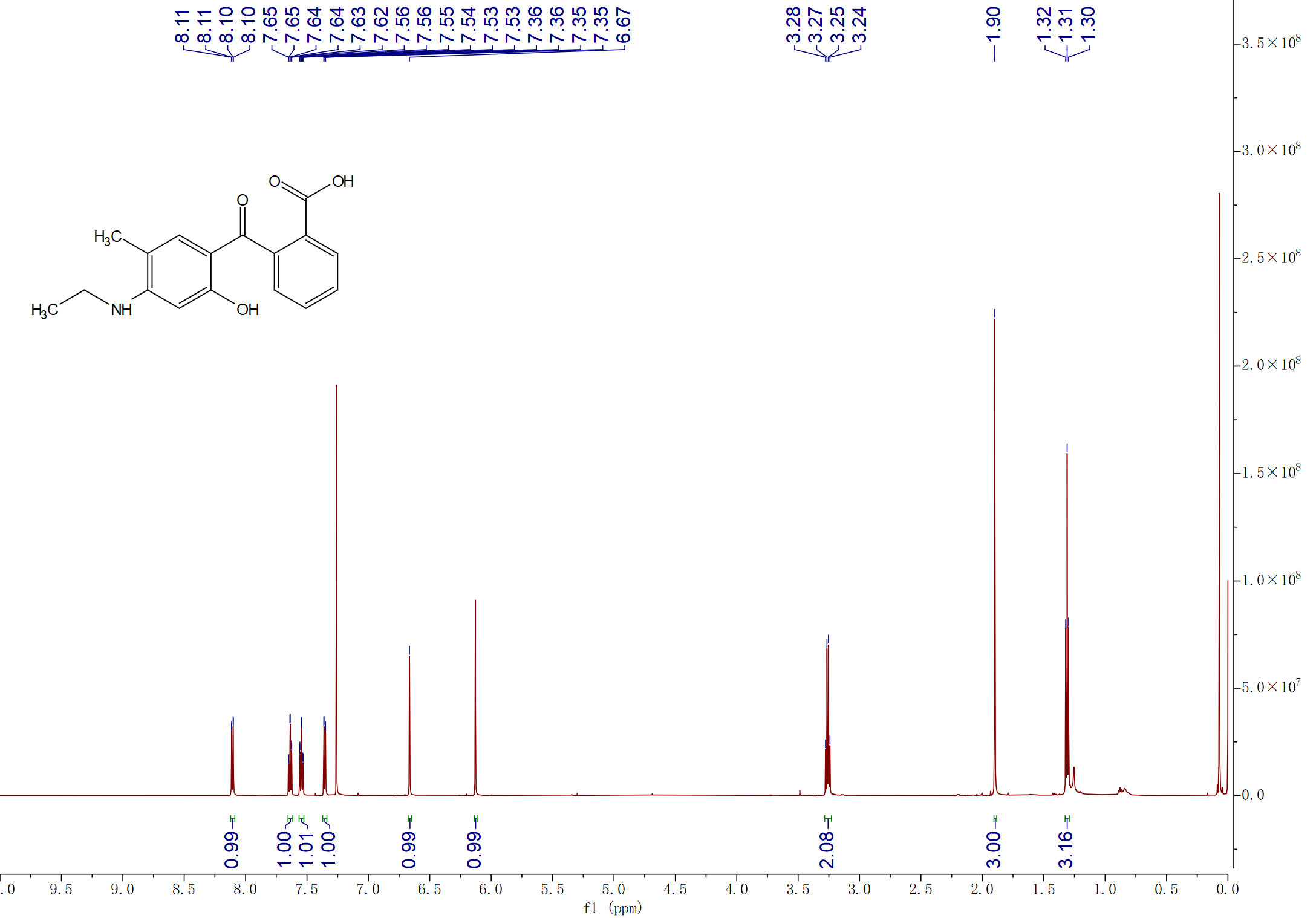


**Figure S10**. ^1^H-NMR spectrum of compound **S3** in CDCl_3_.


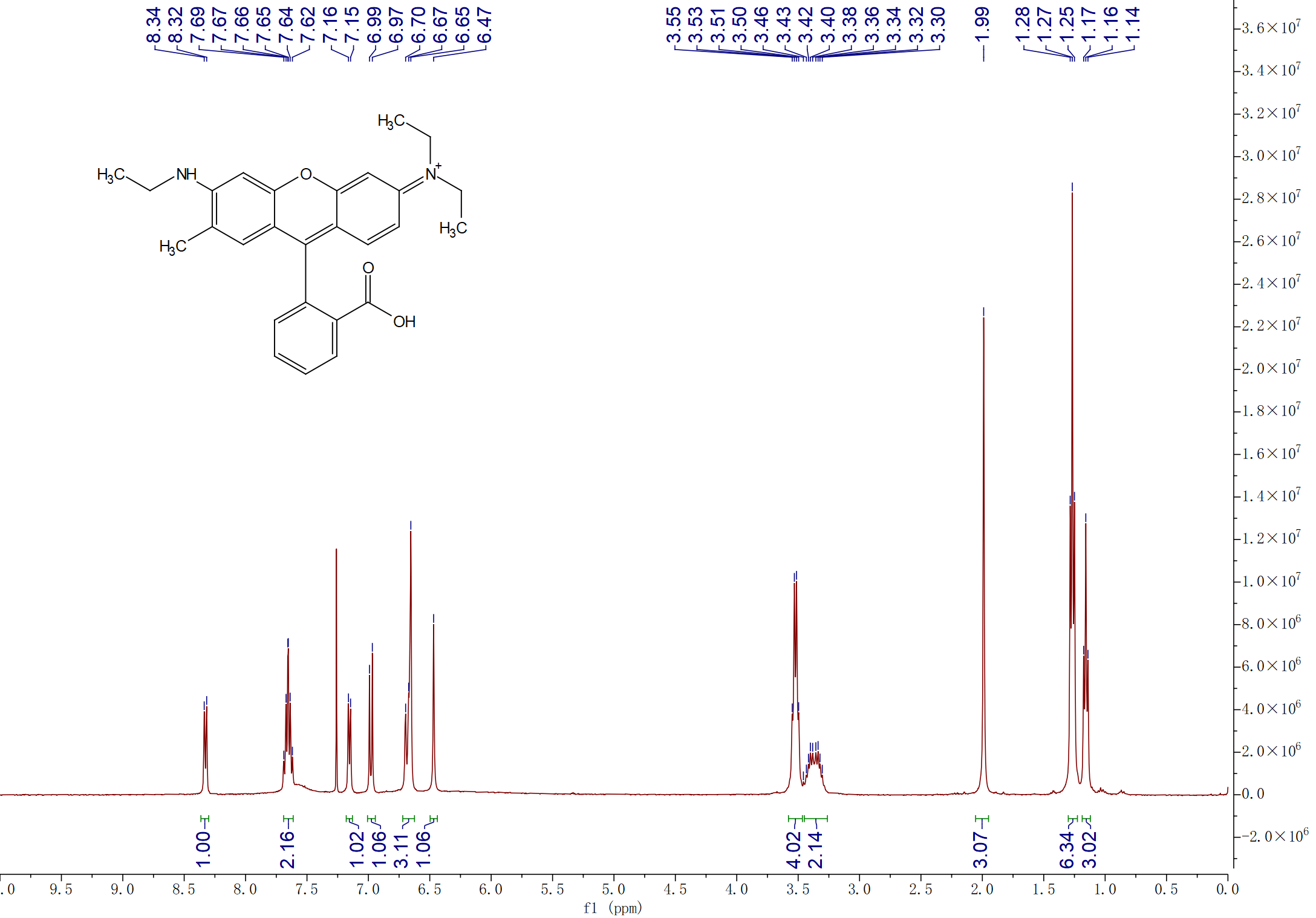


**Figure S11**. ^1^H-NMR spectrum of compound **S4** in CDCl_3_.


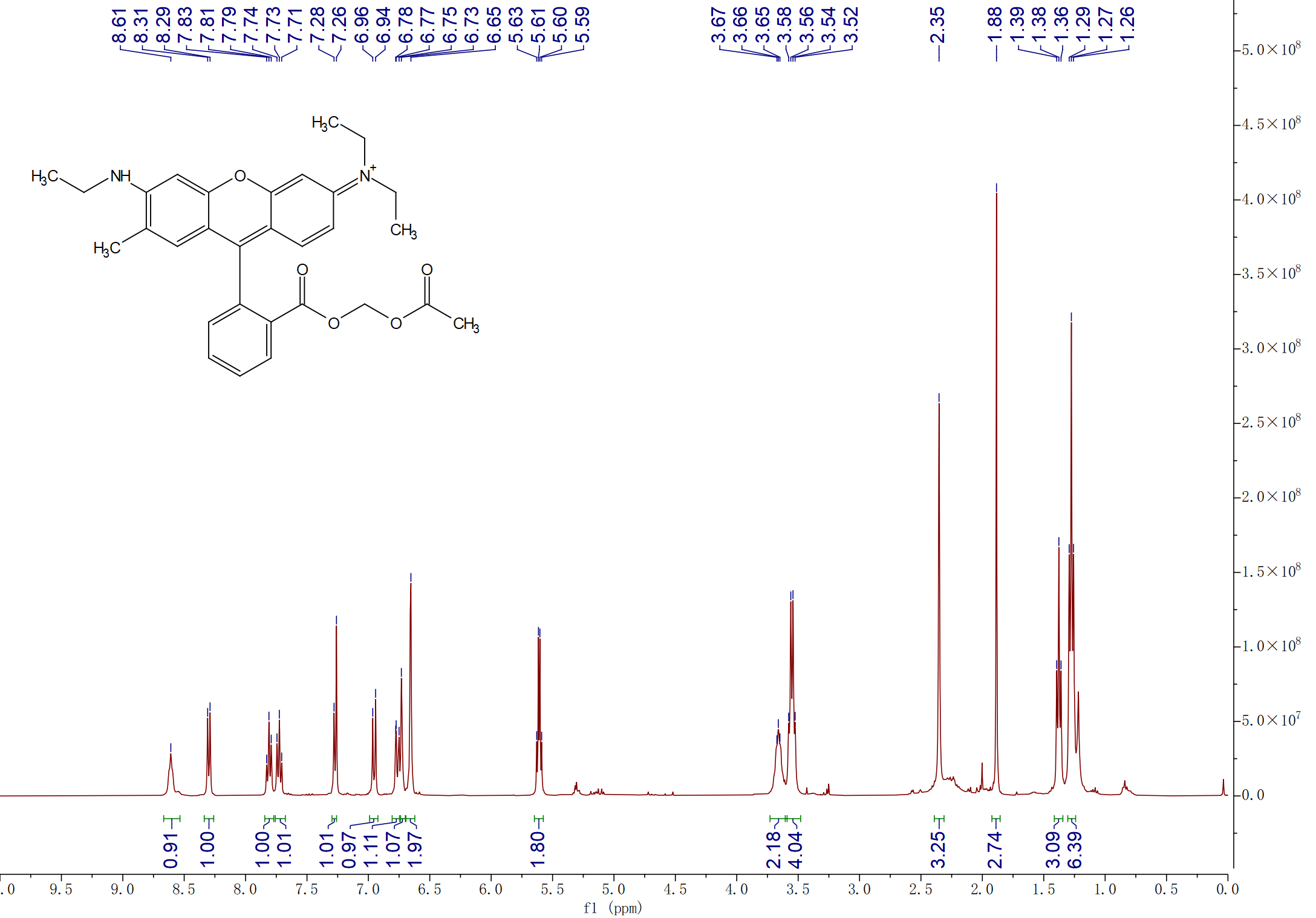


**Figure S12**. ^1^H-NMR spectrum of compound **AM-Rho** in CDCl_3_.


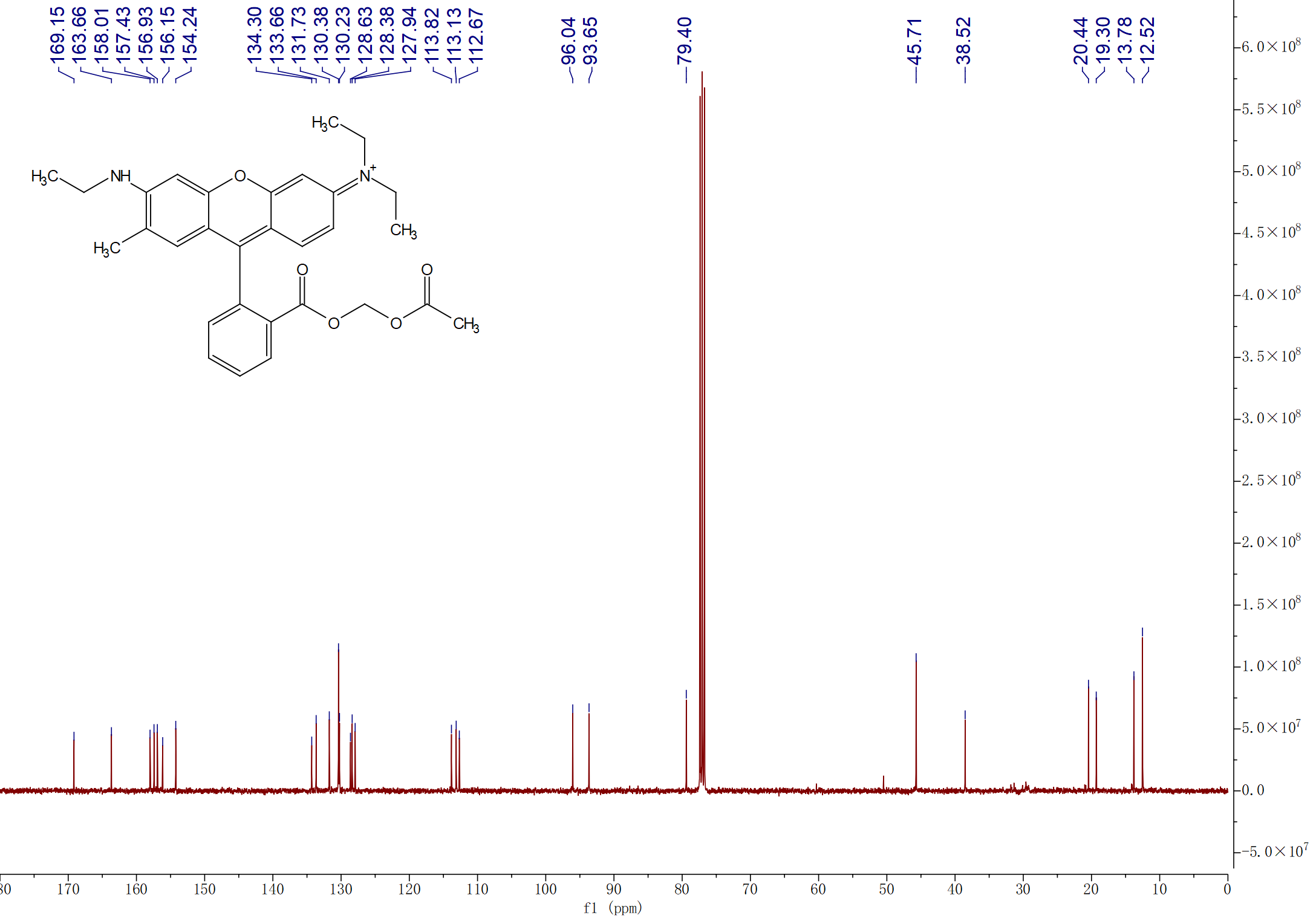


**Figure S13**. ^13^C-NMR spectrum of compound **AM-Rho** in CDCl_3_.


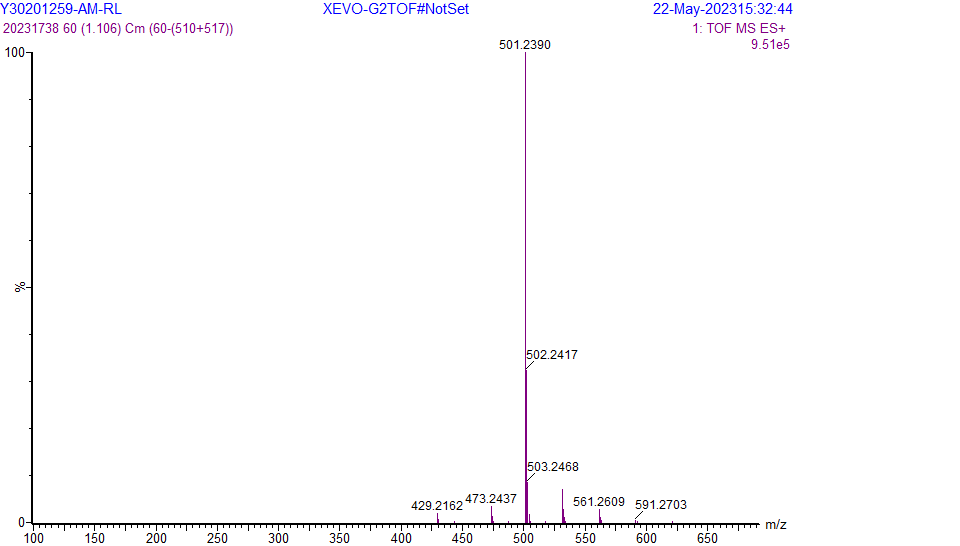


**Figure S14**. HR-MS of compound **AM-Rho**.


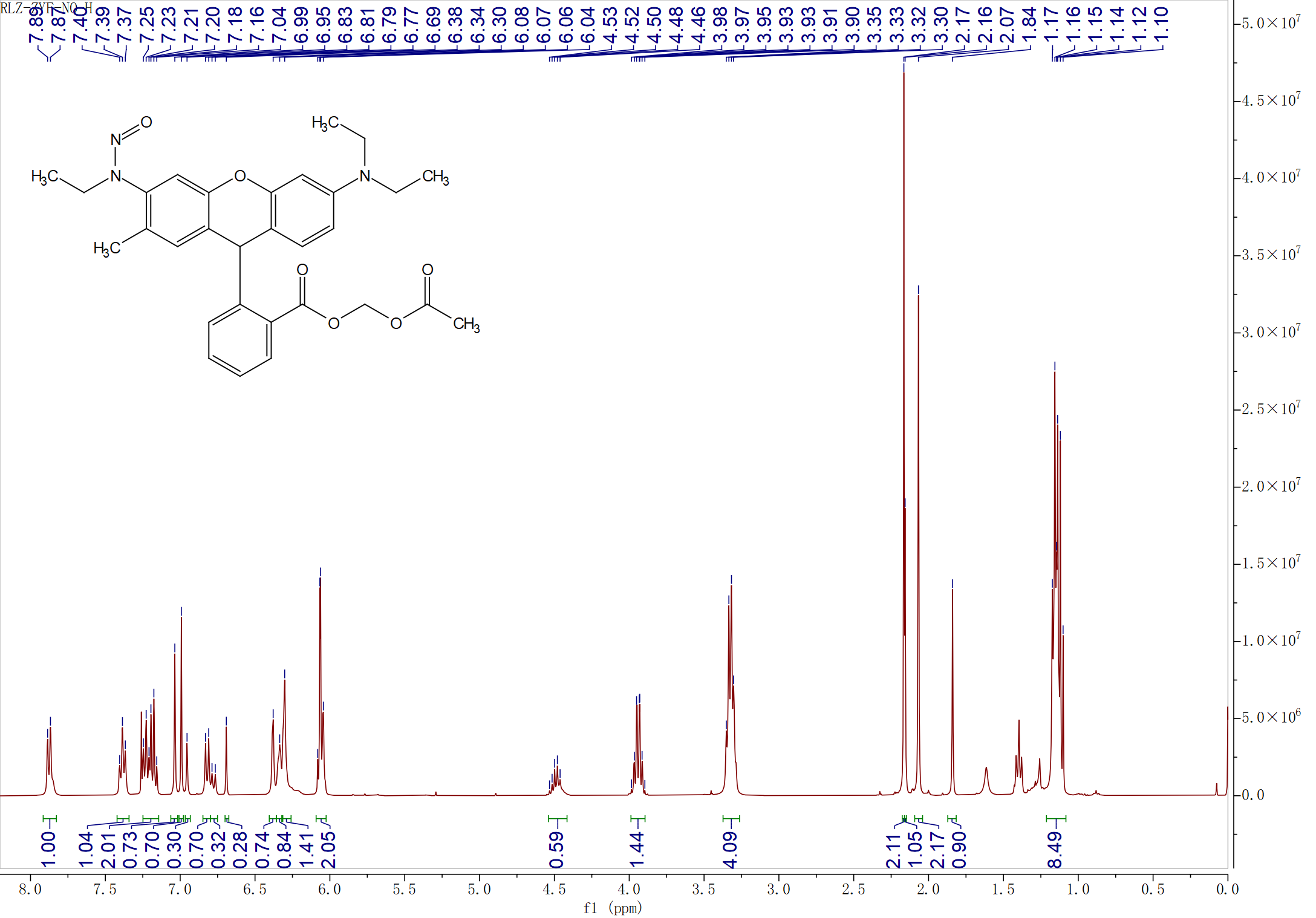


**Figure S15**. ^1^H-NMR spectrum of compound **O-PND** in CDCl_3_.


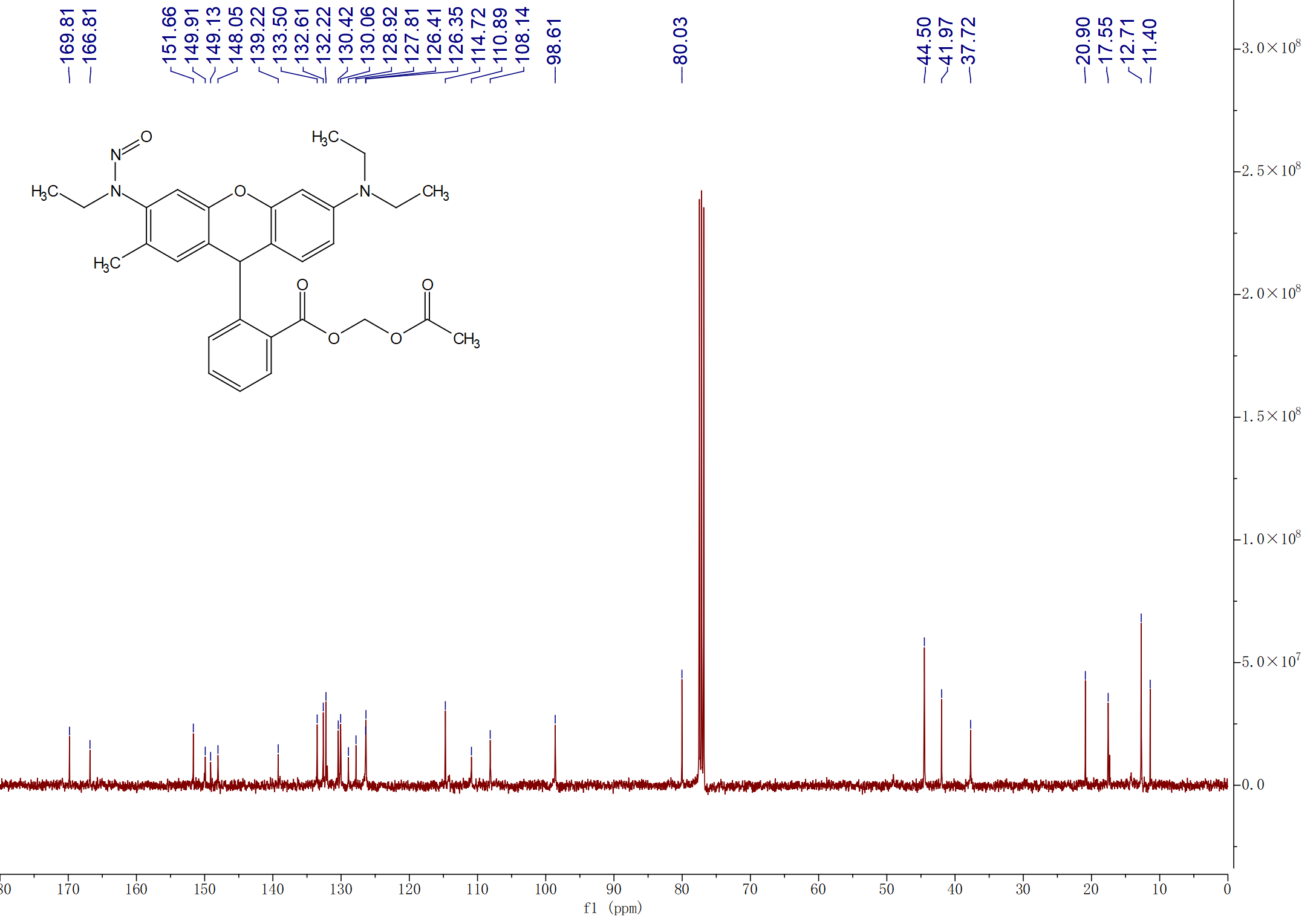


**Figure S16**. ^13^C-NMR spectrum of compound **O-PND** in CDCl_3_.
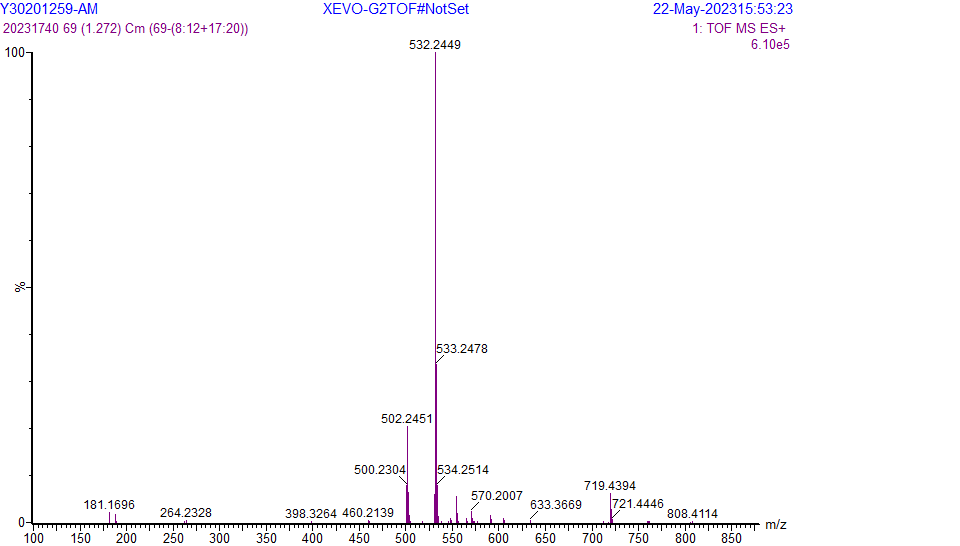


**Figure S17**. HR-MS of compound **O-PND**.


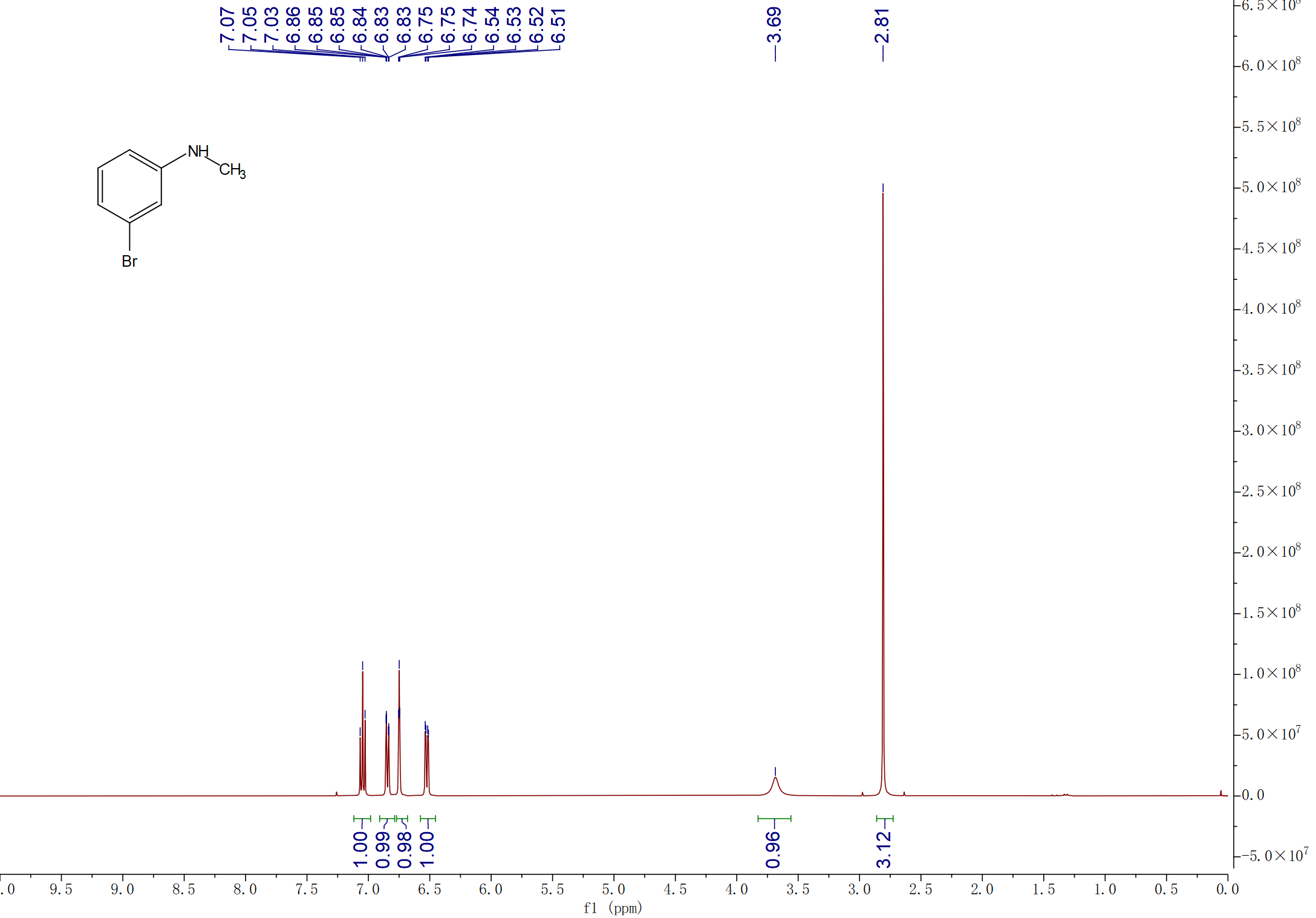


**Figure S18**. ^1^H-NMR spectrum of compound **S6** in CDCl_3_.


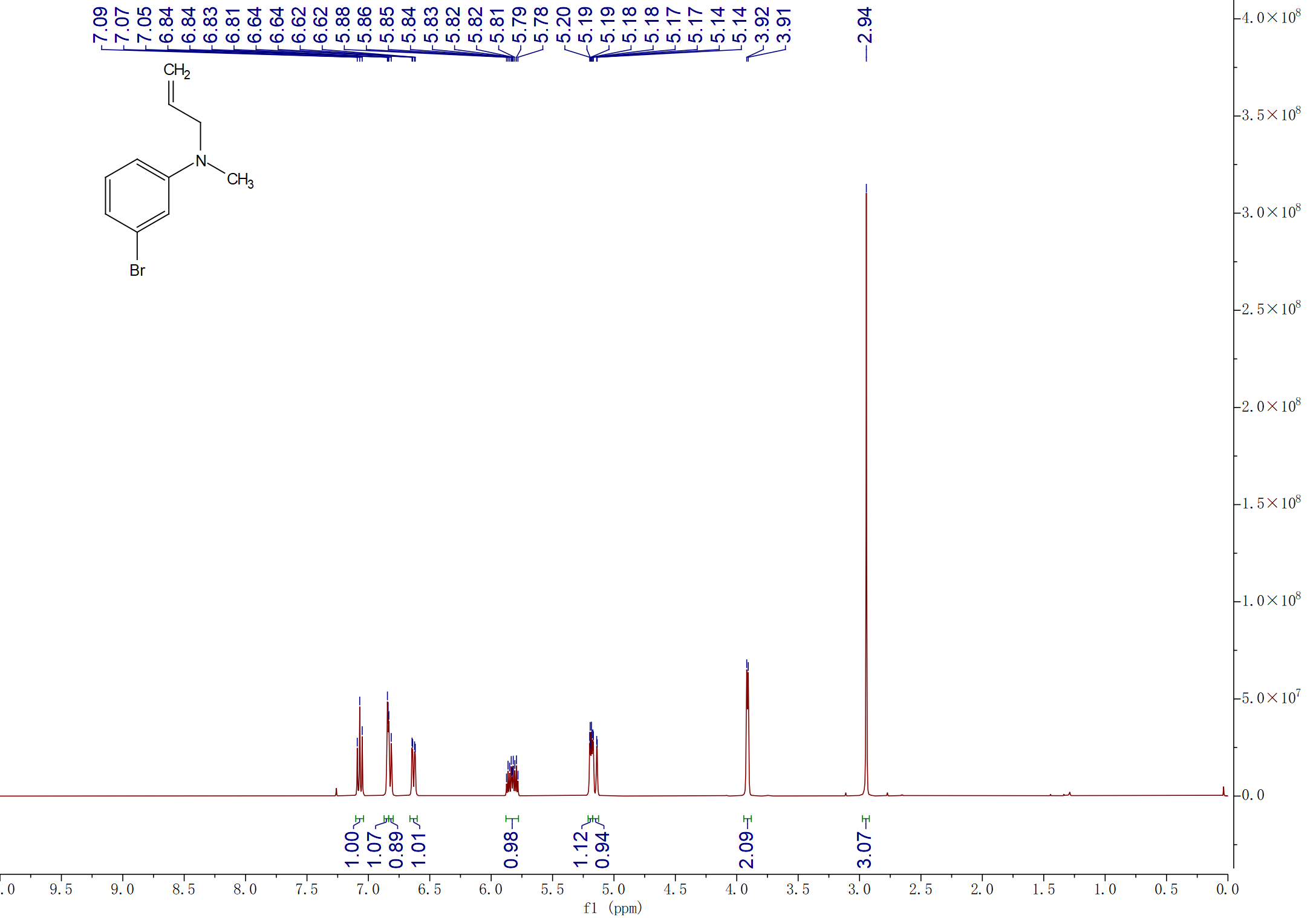


**Figure S19**. ^1^H-NMR spectrum of compound **S7** in CDCl_3_.


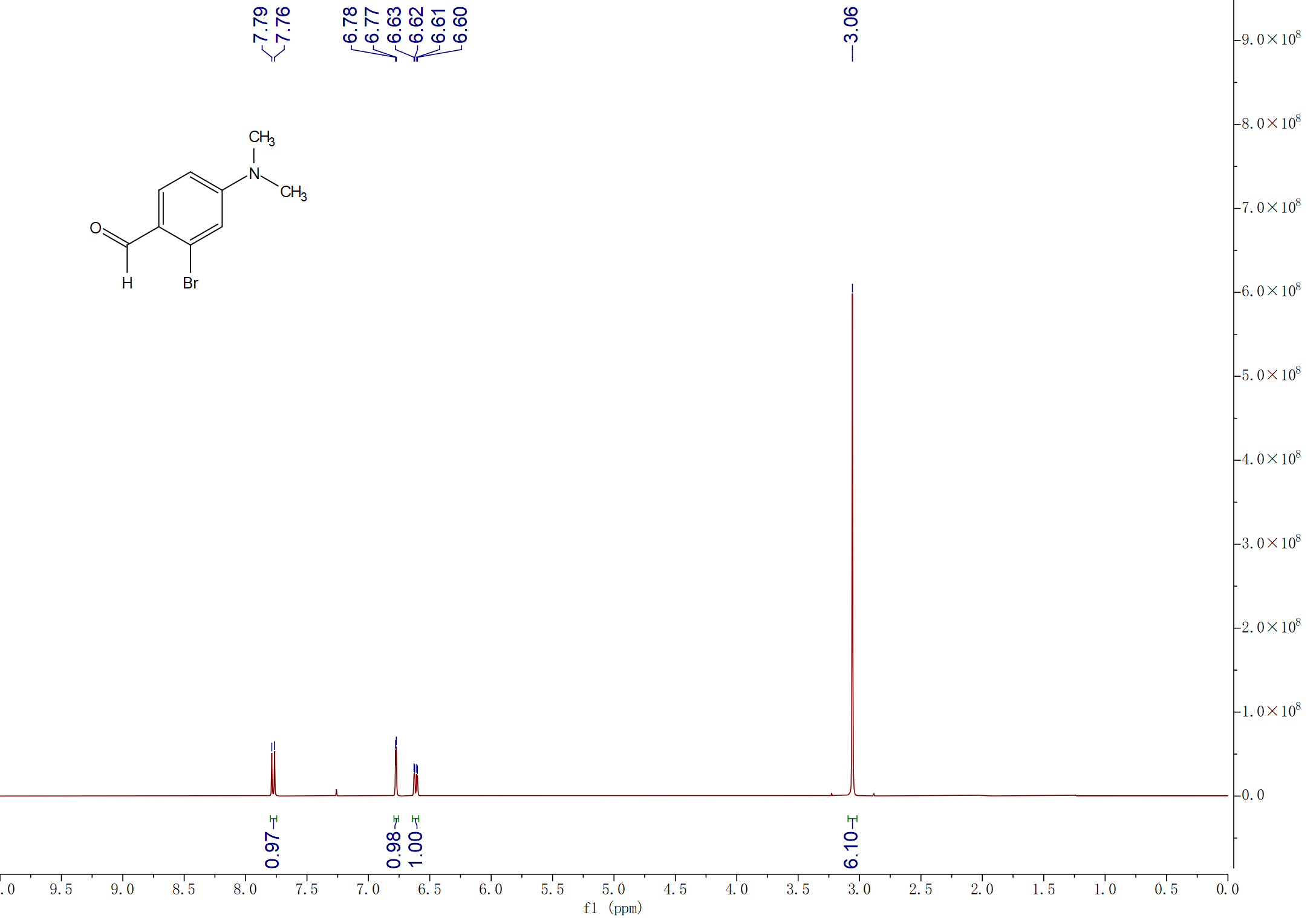


**Figure S20**. ^1^H-NMR spectrum of compound **S9** in CDCl_3_.


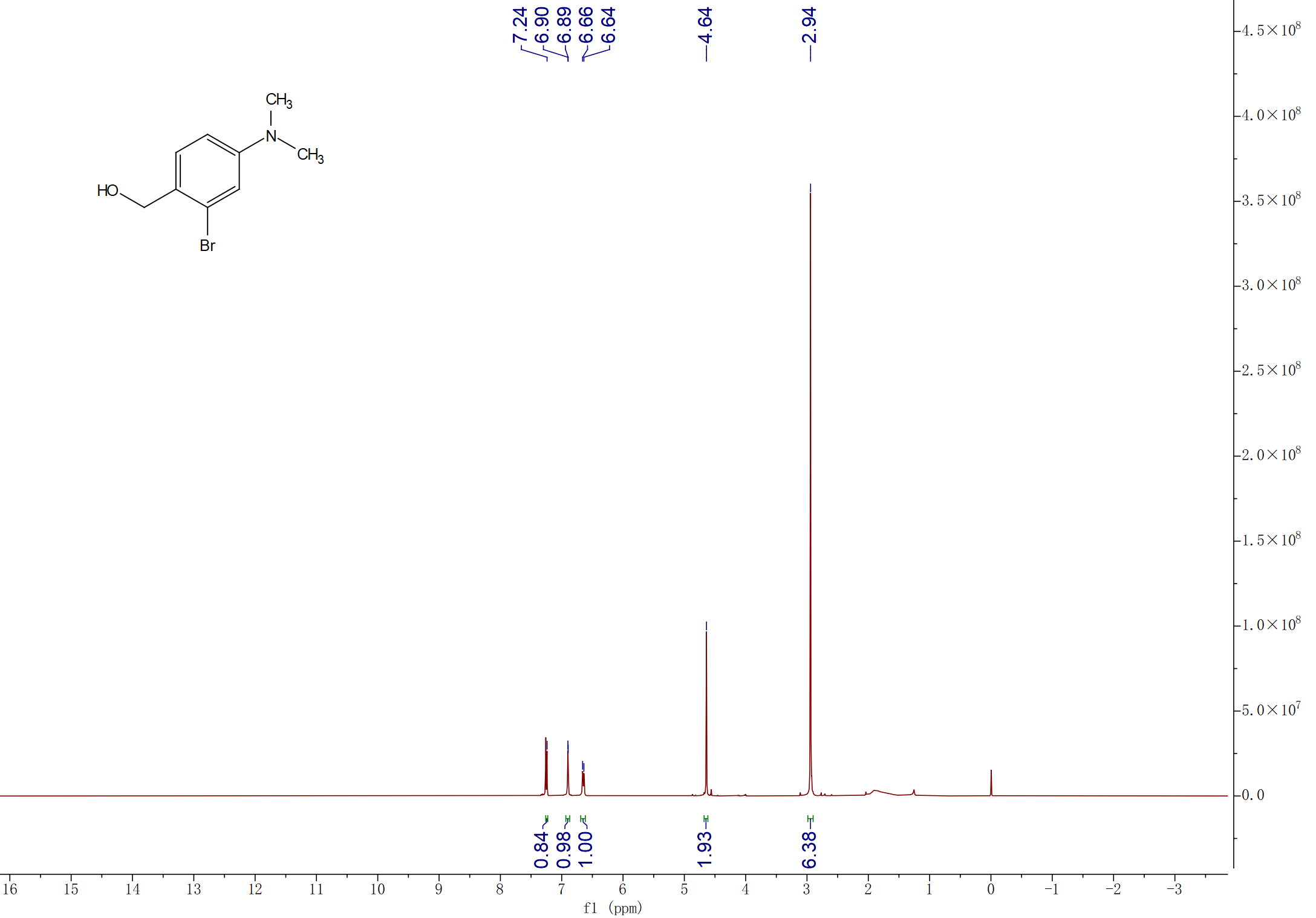


**Figure S21**. ^1^H-NMR spectrum of compound **S10** in CDCl_3_.


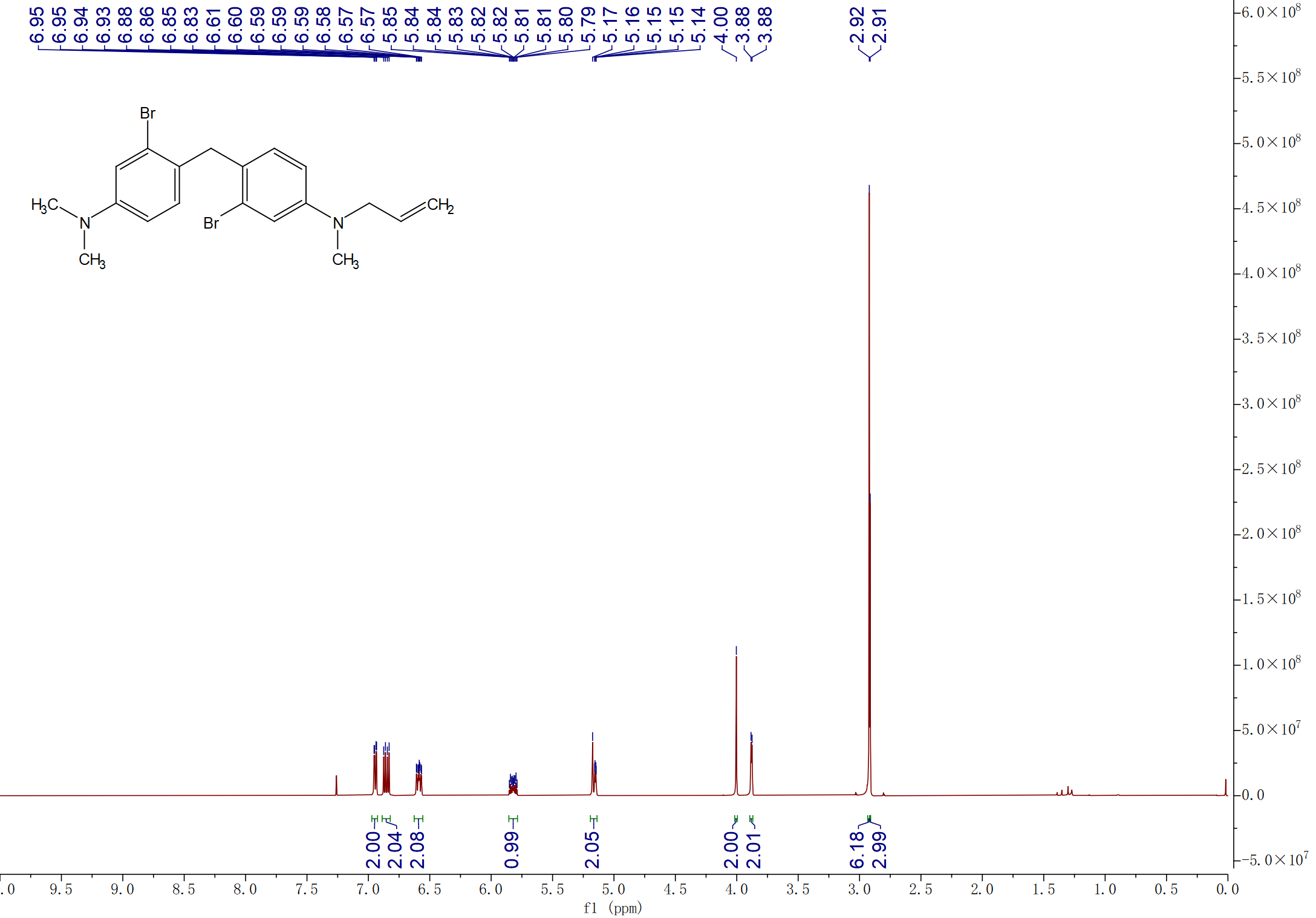
 **Figure S22**. ^1^H-NMR spectrum of compound **S11** in CDCl_3_.


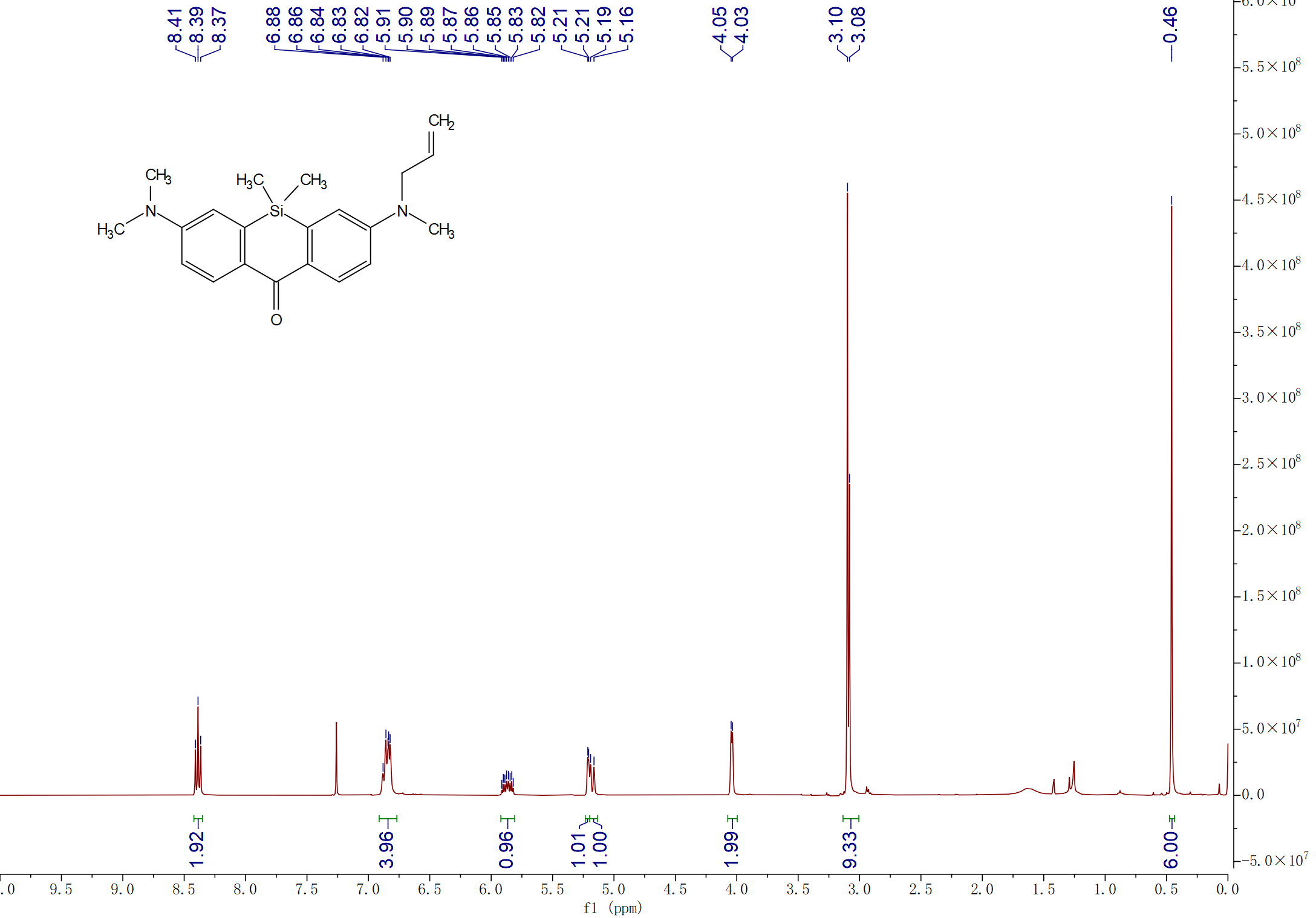
 **Figure S23**. ^1^H-NMR spectrum of compound **S13** in CDCl_3_.


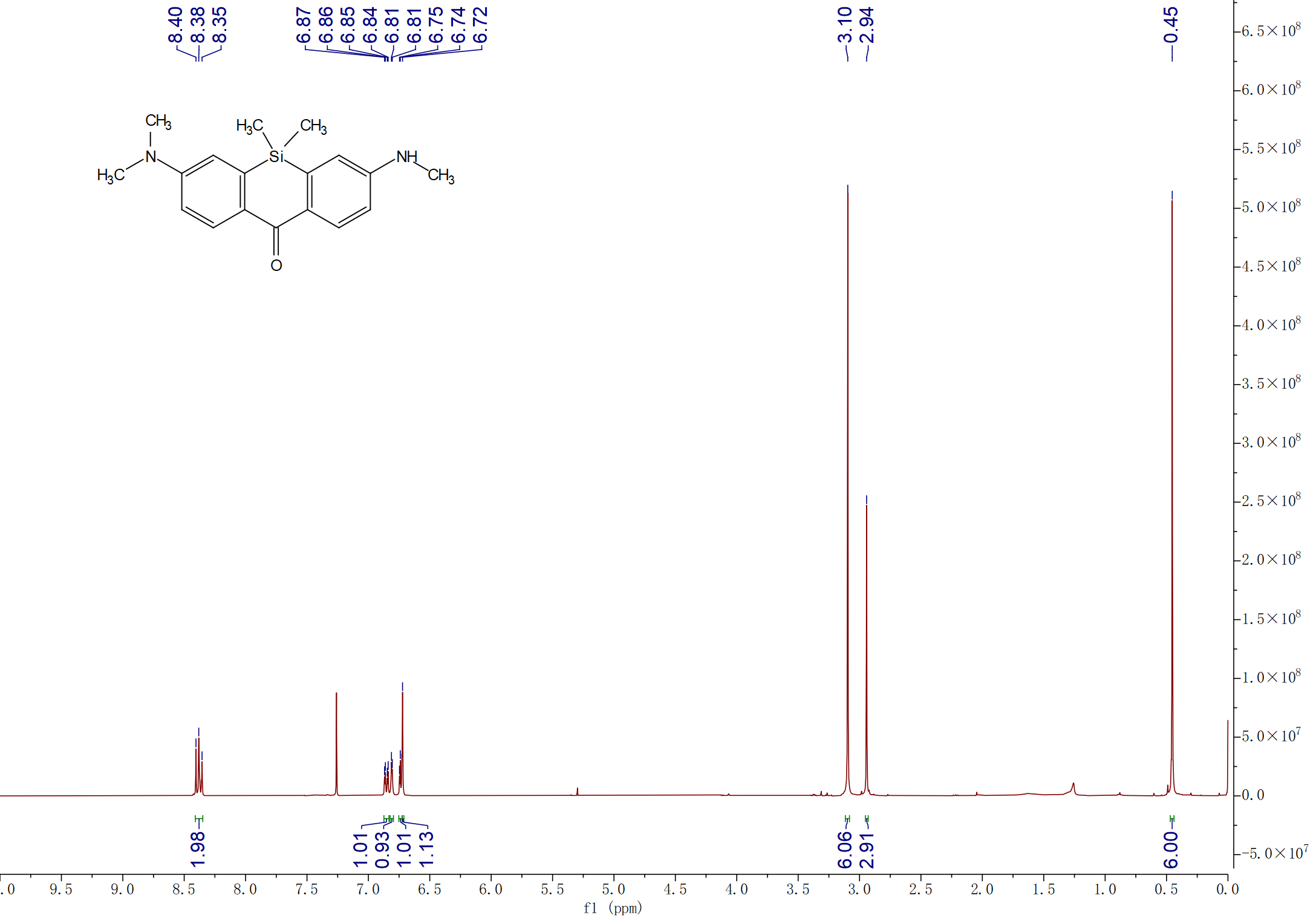
 **Figure S24**. ^1^H-NMR spectrum of compound **S14** in CDCl_3_.


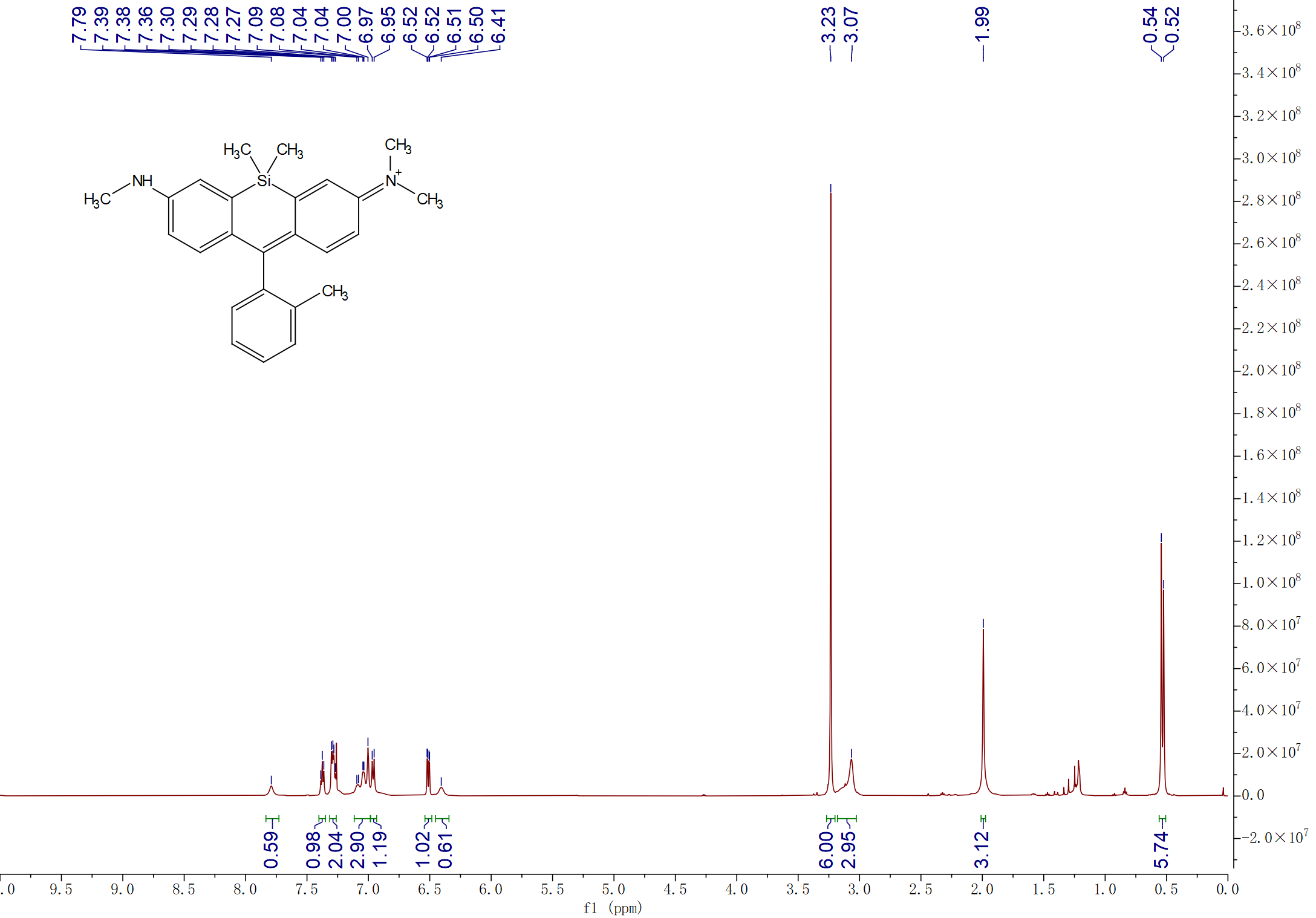


**Figure S25**. ^1^H-NMR spectrum of compound **Si-Rho** in CDCl_3_.


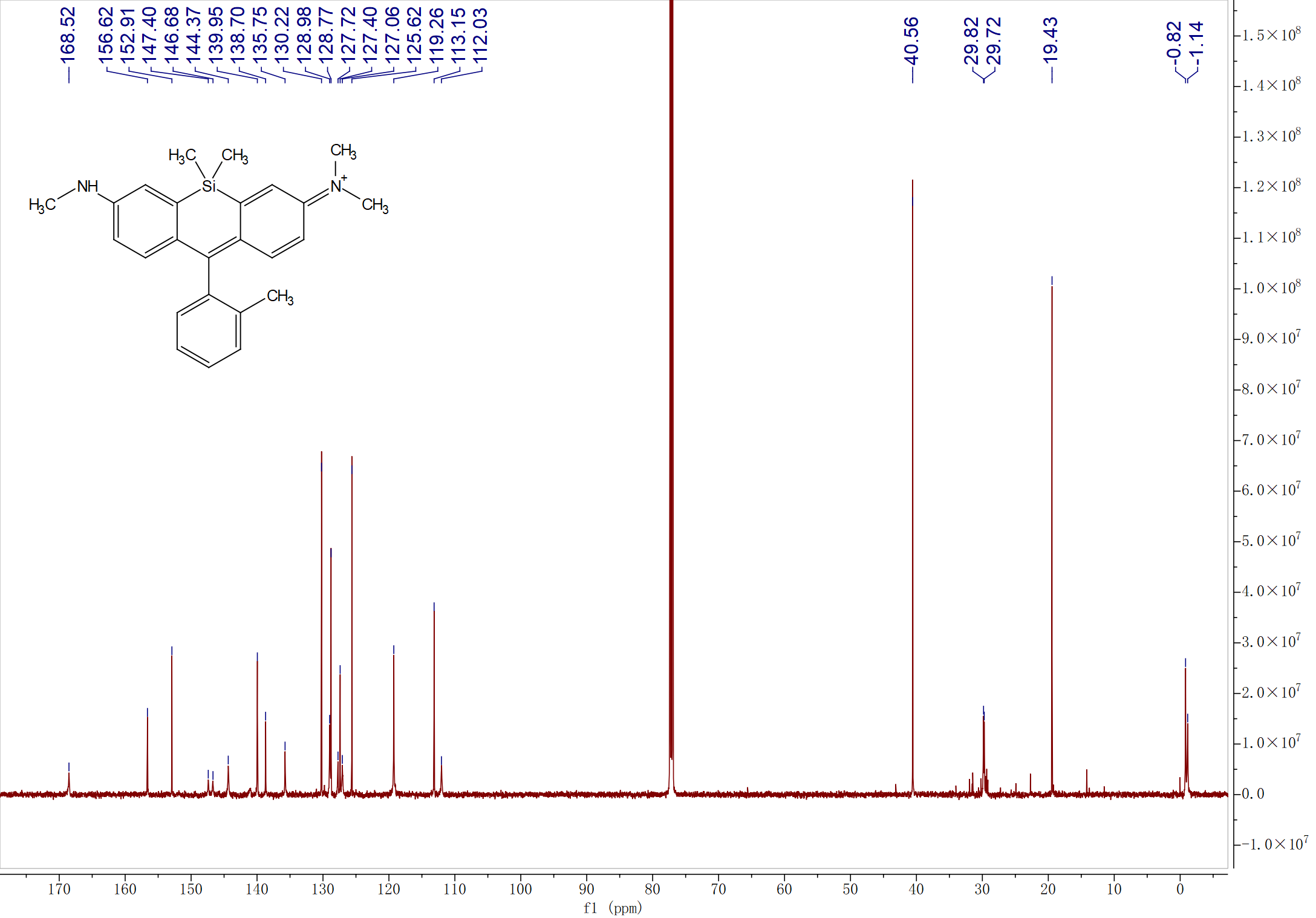
 **Figure S26**. ^13^C-NMR spectrum of compound **Si-Rho** in CDCl_3_.
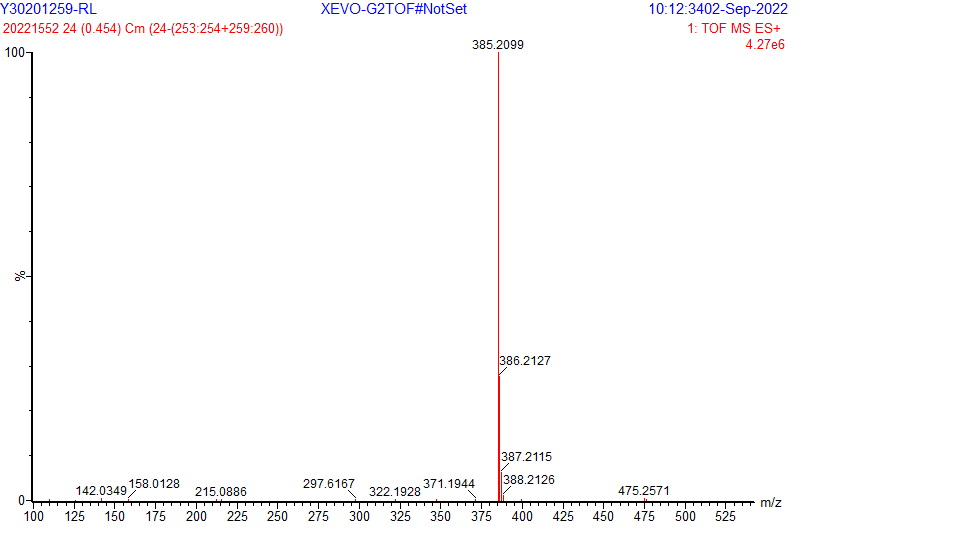
 **Figure S27**. HR-MS of compound **Si-Rho**.


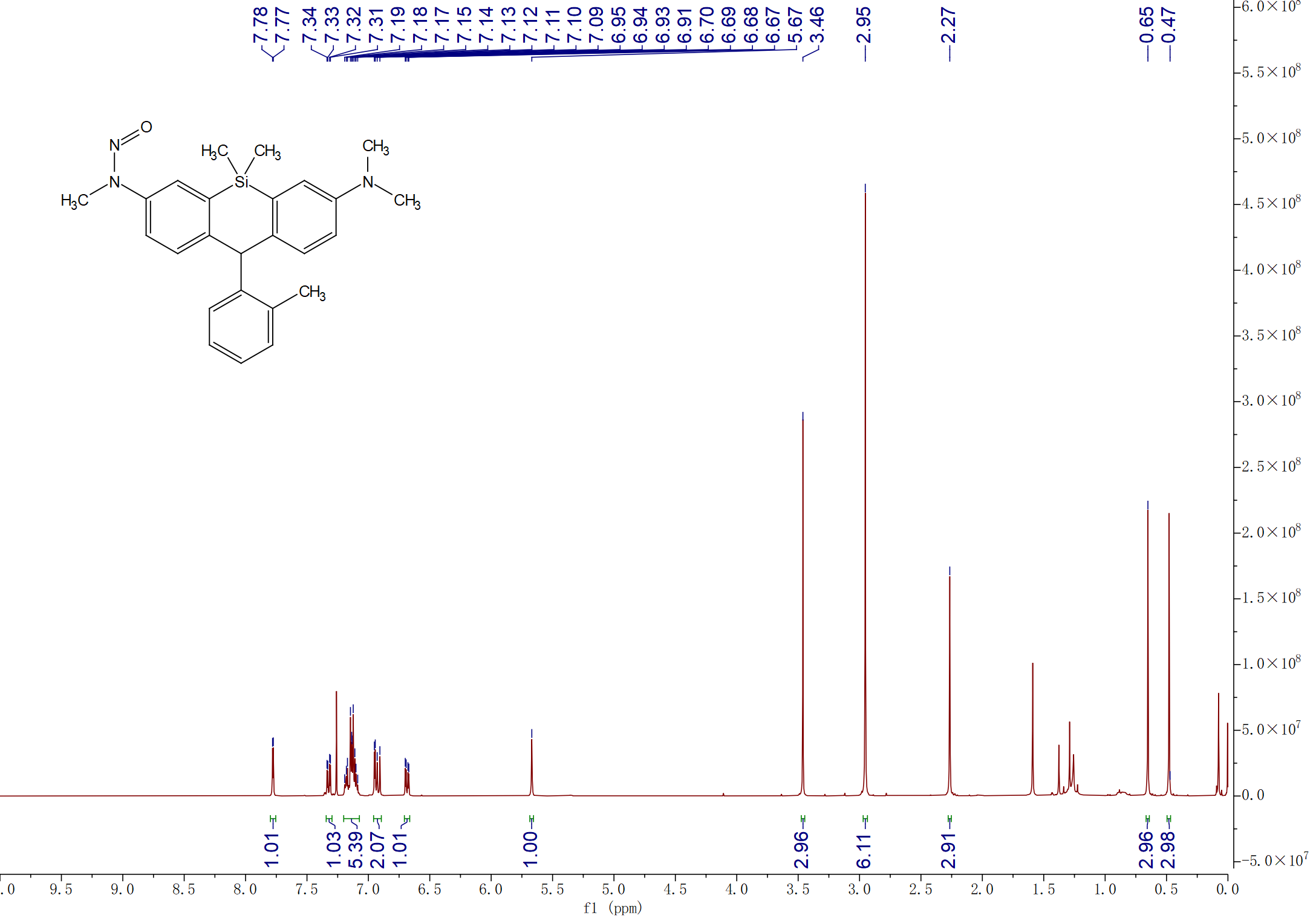
 **Figure S28**. ^1^H-NMR spectrum of compound **Si-PND** in CDCl_3_.


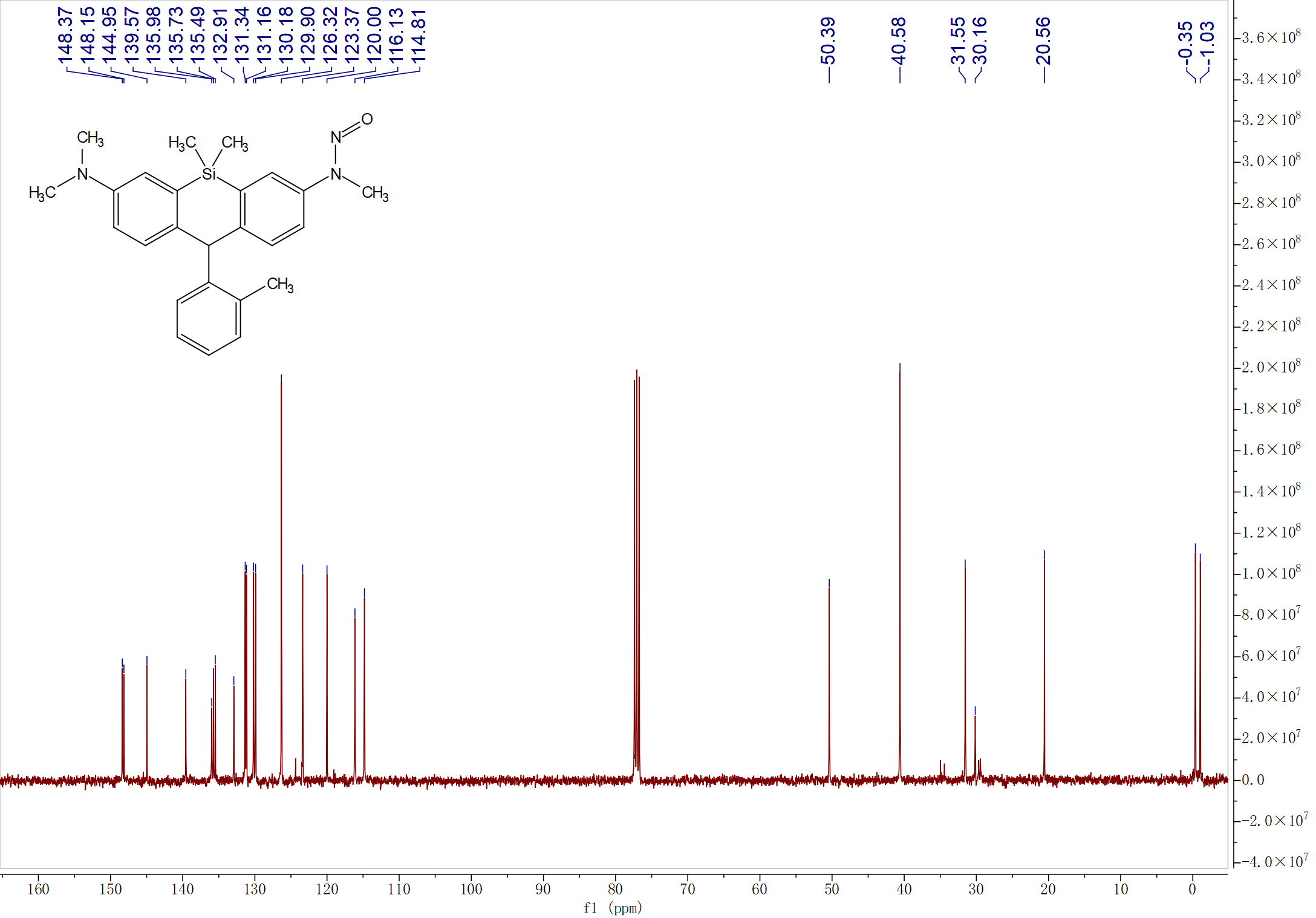
 **Figure S29**. ^13^C-NMR spectrum of compound **Si-PND** in CDCl_3_.


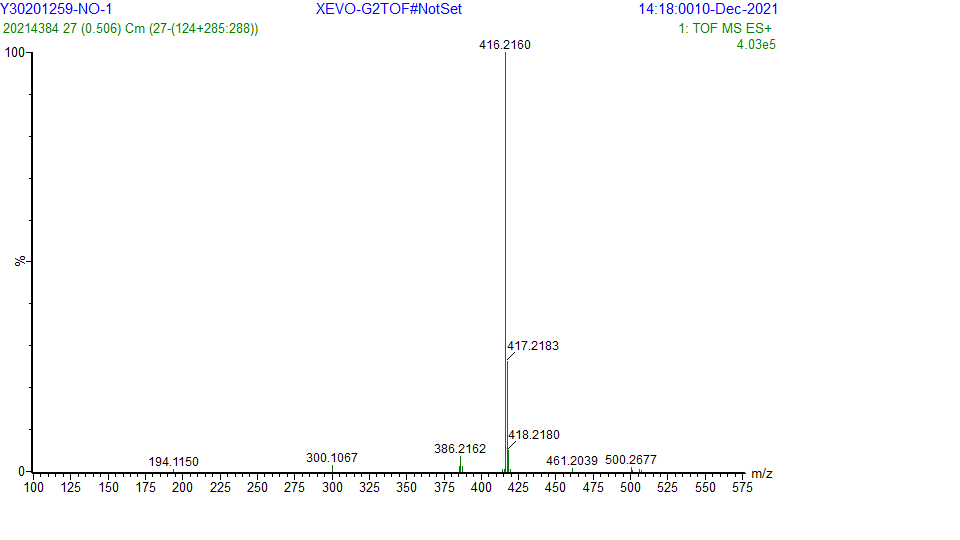


**Figure S30**. HR-MS of compound **Si-PND**.

**References**

1, Zhang, S.; Wang, Q.; Yang, J.; Yang, X.-F.; Li, Z.; Li, H. A Photocalibrated NO Donor Based on N-Nitrosorhodamine 6G upon UV Irradiation. [*Chin. Chem. Lett.*](https://www.x-mol.com/paper/journal/1096) **2019**, *30* (2), 454-456.

2, He, H.; Liu, Y.; Zhou, Z.; Guo, C.; Wang, H.-Y.; Wang, Z.; Wang, X.; Zhang, Z.; Wu, F.-G.; Wang, H.; Chen, D.; Yang, D.; Liang, X.; Chen, J.; Zhou, S.; Liang, X.; Qian, X.; Yang, Y. A Photo-Triggered and Photo-Calibrated Nitric Oxide Donor: Rational Design, Spectral Characterizations, and Biological Applications. *Free Radical Bio. Med.* **2018**, *123*, 1-7.

3, Sasaki, H.; Hanaoka, K.; Urano, Y.; Terai, T.; Nagano, T. Design and Synthesis of a Novel Fluorescence Probe for Zn^2+^ Based on the Spirolactam Ring-Opening Process of Rhodamine Derivatives. *Bioorg. Med. Chem.* **2011**, *19* (3), 1072-1078.

4, Wang, X.; Dong, K.; Yan, B.; Zhang, C.; Qiu, L.; Xu, X. NBS-Mediated Dinitrogen Extrusion of Diazoacetamides under Catalyst-Free Conditions: Practical Access to 3-Bromooxindole Derivatives. *RSC Adv.* **2016**, *6* (74), 70221-70225.

5, Numasawa, K.; Hanaoka, K.; Ikeno, T.; Echizen, H.; Ishikawa, T.; Morimoto, M.; Komatsu, T.; Ueno, T.; Ikegaya, Y.; Nagano, T.; Urano, Y. A Cytosolically Localized Far-Red to Near-Infrared Rhodamine-Based Fluorescent Probe for Calcium Ions. *Analyst* **2020**, *145* (23), 7736-7740.

6, Luo, R.; Wang, Z.; Luo, D.; Qin, Y.; Zhao, C.; Yang, D.; Lu, T.; Zhou, Z.; Huang, Z. Design, Synthesis, and Biological Evaluation of Novel Triazoloquinazolinone Derivatives as SHP2 Protein Inhibitors. [*J. Enzyme Inhib. Med. Chem.*](https://www.x-mol.com/paper/journal/1512) **2021**, *36* (1), 2170-2182.

7, Hanaoka, K.; Kagami, Y.; Piao, W.; Myochin, T.; Numasawa, K.; Kuriki, Y.; Ikeno, T.; Ueno, T.; Komatsu, T.; Terai, T.; Nagano, T.; Urano, Y. Synthesis of Unsymmetrical Si-Rhodamine Fluorophores and Application to a Far-Red to Near-Infrared Fluorescence Probe for Hypoxia. [*Chem. Commun.*](https://www.x-mol.com/paper/journal/7) **2018**, *54* (50), 6939-6942.
